# Economic Impacts of Non-Native Forest Insects in the Continental United States

**DOI:** 10.1371/journal.pone.0024587

**Published:** 2011-09-09

**Authors:** Juliann E. Aukema, Brian Leung, Kent Kovacs, Corey Chivers, Kerry O. Britton, Jeffrey Englin, Susan J. Frankel, Robert G. Haight, Thomas P. Holmes, Andrew M. Liebhold, Deborah G. McCullough, Betsy Von Holle

**Affiliations:** 1 The National Center for Ecological Analysis and Synthesis, Santa Barbara, California, United States of America; 2 Department of Biology, McGill University, Montreal, Quebec, Canada; 3 School of Environment, McGill University, Montreal, Quebec, Canada; 4 Department of Applied Economics and Institute on the Environment, University of Minnesota, St. Paul, Minnesota, United States of America; 5 U.S. Forest Service, Research and Development, Arlington, Virginia, United States of America; 6 Morrison School of Agribusiness and Resource Management, Arizona State University, Mesa, Arizona, United States of America; 7 U.S. Forest Service, Pacific Southwest Research Station, Albany, California, United States of America; 8 U.S. Forest Service, Northern Research Station, St. Paul, Minnesota, United States of America; 9 U.S. Forest Service, Southern Research Station, Research Triangle Park, North Carolina, United States of America; 10 U.S. Forest Service, Northern Research Station, Morgantown, West Virginia, United States of America; 11 Department of Entomology and Department of Forestry, Michigan State University, East Lansing, Michigan, United States of America; 12 Department of Biology, University of Central Florida, Orlando, Florida, United States of America; Smithsonian's National Zoological Park, United States of America

## Abstract

Reliable estimates of the impacts and costs of biological invasions are critical to developing credible management, trade and regulatory policies. Worldwide, forests and urban trees provide important ecosystem services as well as economic and social benefits, but are threatened by non-native insects. More than 450 non-native forest insects are established in the United States but estimates of broad-scale economic impacts associated with these species are largely unavailable. We developed a novel modeling approach that maximizes the use of available data, accounts for multiple sources of uncertainty, and provides cost estimates for three major feeding guilds of non-native forest insects. For each guild, we calculated the economic damages for five cost categories and we estimated the probability of future introductions of damaging pests. We found that costs are largely borne by homeowners and municipal governments. Wood- and phloem-boring insects are anticipated to cause the largest economic impacts by annually inducing nearly $1.7 billion in local government expenditures and approximately $830 million in lost residential property values. Given observations of new species, there is a 32% chance that another highly destructive borer species will invade the U.S. in the next 10 years. Our damage estimates provide a crucial but previously missing component of cost-benefit analyses to evaluate policies and management options intended to reduce species introductions. The modeling approach we developed is highly flexible and could be similarly employed to estimate damages in other countries or natural resource sectors.

## Introduction

Invasive species are widely recognized as among the greatest threats to biodiversity and ecosystem stability worldwide, and they impose serious economic and social costs [1], [2], [3]. Global trade yields enormous economic benefits, but a side effect can be the inadvertent transport of organisms from one region to another [4], [5]. Impacts of invasive species have not been adequately accounted for in trade policy, in part because the economic impacts of invaders have not been reliably quantified. Strategies for internalizing the costs of invaders, including pricing, quarantines and tariffs may be the most effective means of avoiding impacts of invasive species if implemented vigorously [6]. An economic rationale for such efforts requires consideration of projected benefits (economic damages avoided) compared to implementation costs. Thus, quantifying the economic damages caused by biological invasions is critical to informing these strategies.

The few studies that have calculated aggregate costs of invasive species have been useful for drawing attention to the economic significance of biological invasions [7], [8], but they have been plagued with difficulties such as double counting certain costs and failing to account for uncertainty and the ability to substitute one resource for another [9], [10]. The difficulties of conducting rigorous economic analysis are compounded by the scarcity of economic data, which are only available for perhaps 1–2% of invaders [11]. Although most non-native species cause low or intermediate impacts [12], in combination these costs can accumulate. To avoid a downward bias, it is critical to model the entire range of impacts rather than assuming that no damages are caused by species for which economic impacts are unknown.

Despite conceptual challenges, economic assessments of the impacts of non-native species are needed to provide credible information to policy makers and to justify costs associated with management efforts [13]. Decisions must often be made in the absence of complete data but it is important to explicitly identify and address the uncertainty inherent in the data [14]. Risk analyses in general, and Bayesian approaches in particular, offer a coherent means of incorporating uncertainty into decision-making. Specifically, it is possible to integrate across an uncertainty distribution, rather than assuming point estimates are correct or being incapacitated in the face of large uncertainties [e.g.15].

We estimated total direct annual costs of non-native forest insects established in the United States. Forests and urban trees provide important economic and social benefits, as well as ecosystem services [13], [16]. Non-native forest insects often encounter evolutionarily naive, vulnerable host trees and few natural enemies when they arrive in a new habitat. These invaders may kill their host trees or affect tree health, growth or appearance. Our analysis is based on an exhaustive database of non-native forest insects in the continental U.S., which enabled us to standardize the area of analysis and to take advantage of available data.

Our objective is to provide improved cost estimates that policy makers can use to inform decision–making in a framework that can be updated and improved as new data become available. In constructing our approach, we advance previous work in three ways. First, we stratify analyses by insect feeding guild. Pests in the same feeding guild generally cause similar types of damage and often share some biological traits. Moreover, guilds are associated with probable pathways of introduction, and therefore are relevant units for trade policy considerations. Second, we separate analyses by economic cost categories to avoid double counting (such as those federal expenditures which subsidize local expenditures) and to highlight the relevance of invasive forest pests to different sectors of society. Finally, we quantify uncertainty in our estimates to reflect the limits of data used in our models.

## Methods

### Established non-native forest insects

We used a database of 455 non-native phytophagous forest insect species known to be established in the continental United States, compiled using published sources and expert input [17]. While the majority of the 455 species have not caused detectable damage, we identified a subset of 62 species that have been reported to cause noticeable impacts (above background levels) to live forest trees [17, part II of Appendix S1]. We assigned each of the 455 species to a feeding guild based on their dominant or most damaging feeding mode – phloem and wood borers (hereafter borers) (71 species), sap feeders (192 species), foliage feeders (155 species), or other (37 species) [17]. For each of the three main feeding guilds, we identified one high impact “poster pest” that was the most damaging species of its guild to date: emerald ash borer (*Agrilus planipennis* Fairmaire*:* borer), hemlock woolly adelgid (*Adelges tsugae* Annand: sap feeder), and gypsy moth (*Lymantria dispar* L.: foliage feeder) [Appendix S1].

### Economic assessments of “poster pests”

We selected five cost categories for analysis for each poster pest, based on data availability. Cost categories included: (1) federal government expenditures (survey, research, regulation, management, and outreach), (2) local government expenditures (tree removal, replacement, and treatment), (3) household expenditures (tree removal, replacement, and treatment), (4) residential property value losses and (5) timber value losses to forest landowners.

Dead and dying trees reduce the value of homes due to lost aesthetic value, create hazards that must be removed by governments and homeowners, and have lower timber value than healthy trees. Although there are political considerations in the allocation of government funding for surveys, research, and outreach activities related to invasive species, we counted these as costs because they expend resources that could have been used for other public services if those invasive species had not arrived. We restrict our analyses to these five cost categories because they cover a significant fraction of the direct costs of forest pests and because data were available. We recognize that there are other indirect costs, secondary effects, and non-market ecosystem services (e.g., changes in water quality, altered species composition) that can be important. Data for assessing these impacts are scarce, however, and methods for scaling local studies up to the national level have not been developed, which would have potentially compounded the uncertainty of our estimates [Appendix S1]. Thus, our analysis should be viewed as providing a lower bound cost estimate. A management action or policy implementation that is worthwhile based on these available direct costs would certainly be deemed valuable if the full range of possible impacts were known.

We estimated short-run (ten year) economic impacts for each cost category using a partial equilibrium framework in which interactions between costs were not considered and which is appropriate when the short-term linkages between economic categories are weak [18]. All economic impacts reflect changes from a baseline scenario reflecting the absence of economic impacts from the poster pests (see part I of Appendix S1, Tables S1,S2,S3,S4,S5, and Figure S1 for a detailed description of the methods and data sources used to estimate economic impacts). Changes in local government and household expenditures were estimated using a dynamic optimization model that captures the economic trade-offs between protecting tree health and the costs of tree removal and replacement. Changes in property values due to changes in tree health were based on economic welfare estimates obtained from published non-market valuation studies. Changes in timber harvesting levels were based on estimates of timber mortality from non-native forest insects, and mortality induced harvest reductions were small enough to have no impact on timber prices. Changes in federal expenditures were based on historical data, as it was deemed to be infeasible to model the budget decision process.

We chose a ten-year horizon to represent the short-run because: (1) this time span encompasses periodic or cyclical behavior typical in forest pest dynamics, (2) uncertainty is constrained by not extrapolating too far into the future, and (3) shorter time horizons could be greatly influenced by stochastic factors, such as weather, or a particular phase of a pest outbreak. Because each pest is at a different stage of invasion, for each poster pest, we selected a ten-year period that would closely reflect average pest-related damages, management options and costs, and for which data were available or could be projected (Table S1). For each poster pest, we converted estimated impacts to constant 2009 US dollars using a 2% real discount rate. We obtained annual costs by calculating an annuity for our discounted damages over a ten-year time horizon.

For all cost categories except federal government expenditures, we estimated economic impacts using spatial data and dynamic models of infestation extent. We did not sum economic impacts across categories to avoid double counting. For example, double counting could occur between federal and local government expenditures due to transfers between the government bodies; homeowner expenditures and residential property value losses could overlap, because property values capitalize the potential real estate losses, including expenditures on tree removal and treatment. This approach facilitates comparison across guilds, within cost categories, but we caution against adding across cost categories. If data related to the extent of overlap between categories become available in the future, adjusted cost categories could be summed.

### Bayesian modeling of total impacts

**Figure 1:**
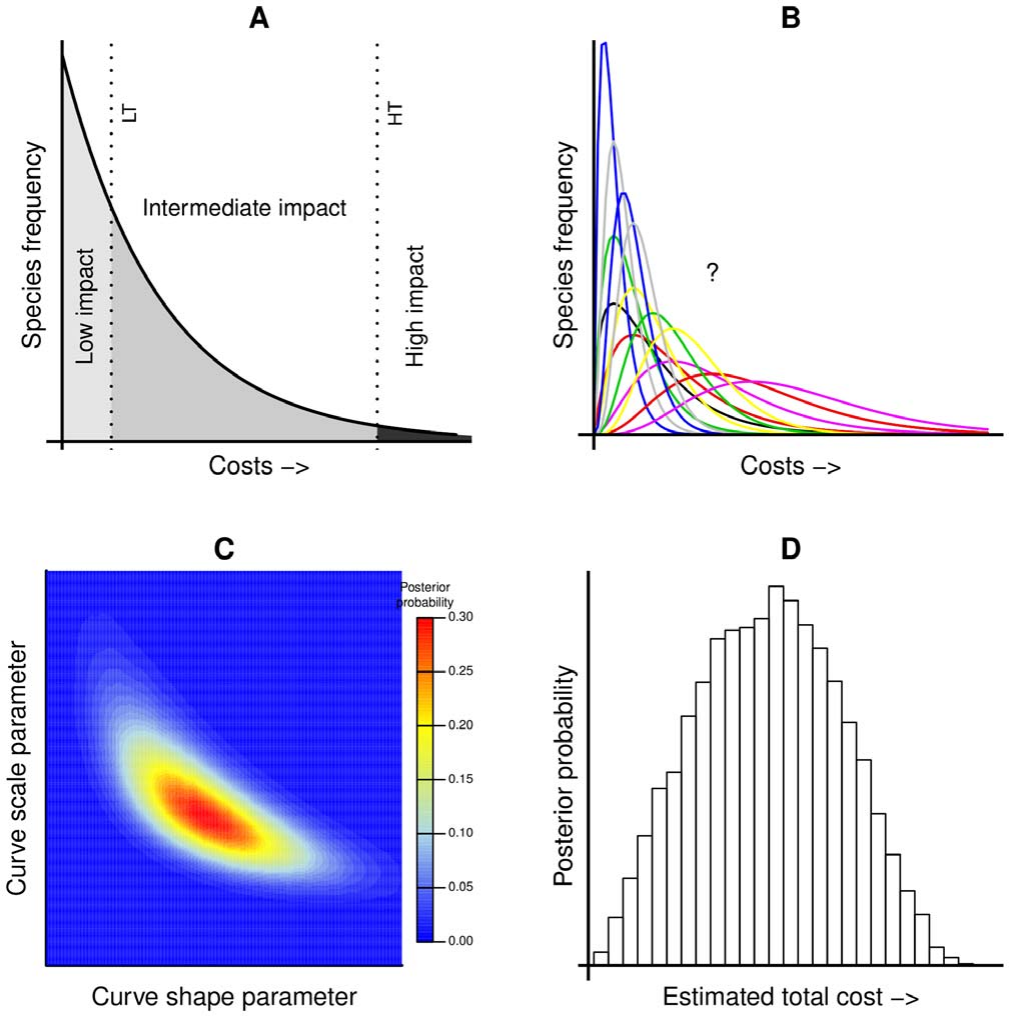
Framework for estimating economic costs of invasive species. A) The hypothetical cost curve is the frequency distribution of annual economic cost caused by invasive species belonging to a feeding guild. The counts of low and intermediate impact species, as well as the low impact threshold (LT) and the level of damages caused by the poster pest (HT) are known; however, the exact shape of the curve is unknown. B) Alternative cost curves. The data are fit using different parameter values, for four alternative models: gamma (illustrated), log-normal, power and Weibull distributions. C) Illustrative Bayesian posterior probability distribution of cost curve parameters. The posterior probability distribution is the relative probability for each cost curve (defined by parameter values). Some cost curves are more likely than others, given the observed data. The posterior probability allows us to consider and incorporate the relative evidence for each cost curve, thereby accounting for parameter uncertainty. This process is repeated for all four models (Weibull, log-normal, gamma (shown), and power function), and then integrated using Bayesian model averaging, which accounts for model uncertainty. The relative probabilities are shown as a heat graph. D) Probability distribution of total annual cost across species in the guild. We converted the cost curves from the Bayesian analysis into a more meaningful metric - total costs from invaders (Appendix S1). Each cost curve and its corresponding total cost has a relative probability of being true given the observed data. The entire process is repeated for each guild and cost category.

For each insect guild and cost category, we estimated the total annual costs (expenditures or losses) across the entire guild and quantified uncertainty given the available data. We began by asserting that there is a frequency distribution of annual costs (the cost curve) (Fig 1A). We assumed that introduced phytophagous species do not have net positive effects on our economic cost categories (cost >0), and that species causing little damage are more common than species that cause intermediate or high impacts, while only a few species cause severe damage [12]. Given these constraints, the possible functional forms that describe the cost curves are limited. Because the exact forms of the cost curves are unknown, we examined several alternative models. We considered 39 parametric families of curves [19], and reduced these to four non-redundant families with appropriate theoretic properties: the gamma, Weibull, power function and log-normal distributions. Although we did not have cost estimates for each species, we used the frequency of species in our database, partitioned into low, intermediate and high damage classes to fit the curve (Fig. 1A). We used expert opinion to define the thresholds between pests that cause low and intermediate costs (Table S6), and our detailed economic analysis of the poster pest for each guild to define the thresholds between intermediate and high costs. By calculating the expected value of each cost curve, and multiplying by the posterior probability, we could then estimate the expected cost of a single species, as well as the total annual cost of all known pests in each guild. Once the shape of the cost curve (and associated uncertainty) has been characterized, any number of derived values of interest can be extracted in a similar way to the expected and total costs. Here, we also present estimates of the probability that a new invader will be more costly than the poster pest (i.e., the area under the curve to the right of the poster pest).

**Figure 2:**
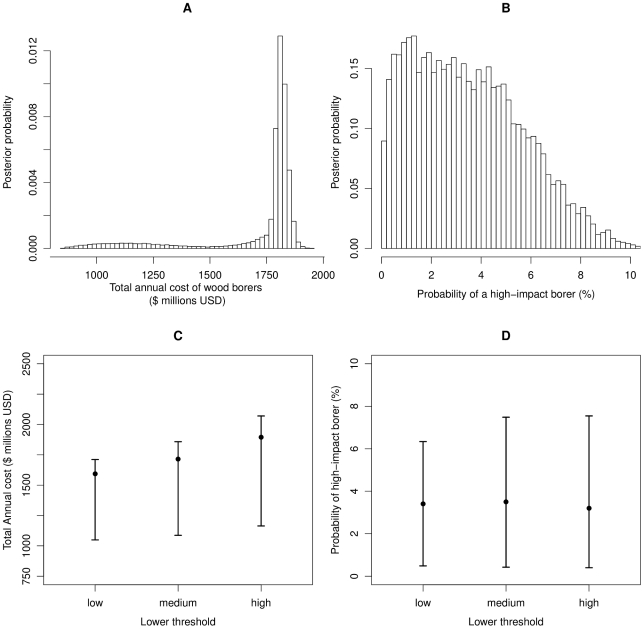
Results for borer feeding guild and local government cost category showing the posterior probability distributions of a) estimated total annual cost of all known borer species, b) probability that a newly introduced borer will cost local governments as much or more than the poster pest (emerald ash borer). Panels c) and d) show the low sensitivity of the posterior predictions to alternative specifications of the low impact threshold (LT) which was based on expert opinion (Appendix S1). Shown are alternative specifications for total annual guild costs (c) and probability of a high impact borer (d) across two orders of magnitude, where low, medium and high costs are defined as 150, 1,500 and 15,000 annual USD, respectively (Table S6).

We accounted for uncertainty by quantifying variability among species (the cost curve; Fig. 1B), parameter uncertainty (Bayesian analysis; Fig 1C), and model uncertainty (Bayesian model averaging across the four families of curves; Fig 1D, Fig 2 A,B). Further, because they were classified by expert opinion, we performed sensitivity analysis on the lower threshold, spanning the threshold value by two orders of magnitude (Fig. 2C,D, S2, S3). As new data become available in the future, damage estimates can be readily updated to re-evaluate total cost estimates. We report the Bayesian expectations in the text (i.e., the mean of the Bayesian posterior distribution), as well as the 90% credible intervals in Table 1. For further details of the framework and complete model specification, see part III of Appendix S1.

**Table 1:** Annualized damage in U.S. $1,000,000 associated with each guild and cost category.

Guild	Federal Government Expenditures	Local Government Expenditures	Household Expenditures	Residential Property Value Loss	Forest Landowner Timber Loss
BORERS (N = 71, N_i_ = 14)					
Poster: emerald ash borer damages ($10^6^)	38	850	350	380	60
Total damage ($10^6^)	92 [62–97]	1700 [1100–1900]	760 [460–820]	830 [510–900]	130 [81–150]
P>poster (%)	3.5 [0.47–8.1]	3.4 [0.43–7.2]	3.4 [0.39–7.6]	3.3 [0.41–7.8]	3.3 [0.42–7.5]
SAP FEEDERS (N = 192, N_i_ = 19)					
Poster: hemlock woolly adelgid damages ($10^6^)	4.3	66	44	100	1.1
Total damage ($10^6^)	14[6.6–15]	170 [85–190]	130 [62–140]	260 [130–290]	4.2 [2.1–4.6]
P>poster (%)	1.1 [0.14–2.8]	1.1 [0.12– .8]	1.1 [0.13– .1]	1.0 [0.14–2.5]	1.1 [0.15–2.9]
FOLIAGE FEEDERS (N = 155, N_i_ = 25)					
Poster: gypsy moth damages ($10^6^)	33	50	46	120	4.6
Total damage ($10^6^)	110 [52–120]	170 [75–180]	160 [72–180]	410 [190–450]	18 [8.2–20]
P>poster (%)	1.3 [0.21–3.4]	1.4 [0.20–3.6]	1.2 [0.21–3.1]	1.6 [0.22–4.7]	1.3 [0.23–3.4]

The poster pest damage was calculated for each cost category (See Appendix S1 for detailed methods). We report the posterior mean of total damage (see Figure 1) and the probability that a newly introduced pest will be as damaging or more damaging than the poster pest for that cost category (P>poster). N is the total number of species in the guild and N_i_ shows the number of intermediate pests in each guild. The 90% Bayesian credible intervals are in brackets.

## Results

Pests from the borer guild, which often arrive on wood packaging materials, generally exacted the highest total costs across categories (Table 1). At an estimated $1.7 billion in local government expenditures and approximately $830 million in lost residential property values each year, borers' economic impacts were several times greater than impacts from other guilds. Of the three guilds, borers were represented by the fewest species, but a high proportion of them (20%) are damaging [17]. Furthermore, integrated across the uncertainty distribution, the probability that the next species to invade will cause damages at least as great as the poster pest was substantially higher (3.4%) for borers than for the other guilds, although Bayesian credible intervals overlap for the average probability (Table 1).

Sap feeders accounted for the largest proportion of the insects in our database, but relatively few cause tree mortality or substantial damage (Table 1). The high frequency of sap feeder invasions may be attributed in part to the historical trade in live plants, a pathway for introduction of these insects [20]. Of the three guilds, sap feeders caused the least timber value loss; the timber value loss caused by sap feeders was less than 5% of that caused by borers. Efforts to control or manage sap feeders received the fewest federal dollars ($14 million annually), although they caused substantial losses in real estate values - approximately $260 million per year.

Foliage feeders, also frequently introduced with live plants, were almost as abundant in the dataset as sap feeders. Costs associated with foliage feeders were substantially lower than costs associated with borers for all categories except annual federal expenditures, which were slightly greater ($110 million for foliage feeders and $92 million for borers) (Table 1). Foliage feeders were estimated to cause approximately $410 million per year in lost property value. Foliage feeders, such as gypsy moth, typically cause mortality only if consecutive years of severe defoliation occur or under exacerbating circumstances such as drought, which is reflected in the lower costs of this guild.

## Discussion

Government officials, resource managers and property owners routinely make decisions about trade or regulatory policies and about whether or how to manage an invasive forest pest. These decisions should ideally consider the costs of specific actions as well as the benefits to be gained by them. This process is challenging, particularly given the limited information about the current or potential costs of pest impacts. We identified costs likely to be incurred by different societal sectors that can be used in such cost-benefit analyses and the damages associated with major guilds of insects, while incorporating uncertainty to the extent possible.

Our analysis indicates that the cost of non-native forest insects is largely borne by homeowners and municipal governments, large constituencies that may not be adequately considered in most analyses [10]. For all guilds, local government expenditures and residential property value losses were the two highest cost categories. Household expenditures were also high, which was partially reflected in property value loss. The costs of tree removal, replacement and treatment outweighed the costs of federal government containment programs by at least one order of magnitude.

In contrast, we found that timber value losses are relatively modest, often an order of magnitude lower than local government expenditures. This reflects timber values of the tree species attacked by the poster pests. Timber mortality induced by poster pests constituted a small proportion of overall timber harvest volumes across tree species, so we assumed timber supply curves were unaltered. However, we recognize that future biological invasions could have more severe impacts on timber species. In the case where a biological invasion causes catastrophic mortality of valuable timber species, timber buyers and forest owners with non-impacted stands face changes in economic welfare due to market price impacts [21], [22]. A previous estimate of nation-wide economic impacts of non-native forest insects based on timber losses was $2.1 billion annually. However, this estimate assumed a reduction in gross domestic product of timber-related industries and did not consider substitutability [10].

In addition, our analysis highlights the importance of borers, which were consistently the most costly insect feeding guild. The best estimate of costs to local governments, integrating across the uncertainty distribution, was $1.7 billion per year. Despite the presence of substantial uncertainty, even lower bound estimates revealed considerable costs. For instance, phloem and wood borer damage is expected to cost local governments at least $1.1 billion per year but could cost as much as $1.9 billion per year. Indeed, the effect of the borer guild on local governments dwarfed all other cost categories – most by over an order of magnitude.

The relatively high cost of borers in general may not be surprising given that several borers can cause mortality of their host trees. But this finding is particularly troubling because of the dramatic increase in new detections of established borer species in the last 30 years, coinciding with increased use of wood packaging material, which can transport these organisms [17], [23]. Borers accounted for 56% of forest insect invaders detected from 1980–2006, compared to just under 11% before 1930 [17]. Put another way, by integrating the results from our study, there is a 32% risk that a new borer that is as damaging as or more costly than the emerald ash borer will invade in the next 10 years [calculated as (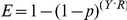), where p is the probability of each introduced pest being more costly than emerald ash borer, Y is the number of years and R is the annual rate of introduction; YR is the expected number of borers introduced. See Appendix S1, eq. 13]. However, if recent international standards which target pathways of introduction such as wood packaging materials (e.g. ISPM-15) are effectively implemented, this introduction rate may be reduced [24]. Although our calculations address the probability that a new poster pest will become established, theoretically, our approach can be extrapolated to any damage level of interest to researchers or policy makers.

The similarity of federal government expenditures for borers and foliage feeders was notable, given that borers (e.g. emerald ash borer and Asian longhorned beetle) generally cause more tree mortality than foliage feeders (e.g. gypsy moth). Some sap feeders also cause localized host mortality (e.g. hemlock woolly adelgid and balsam woolly adelgid), but federal expenditures were almost seven-fold lower for this guild of pests than for defoliators or borers. Federal allocations may reflect factors such as the temporal or spatial extent of a pest, its impacts, the availability of regulatory and management options and external pressures from stakeholders. Hemlock woolly adelgid, for example, may have lower federal costs because it is not regulated. Cost benefit analyses have demonstrated the economic value of efforts such as the gypsy moth “Slow the Spread” program, where government activities prevent or defer costs that would otherwise likely be borne by property owners or municipalities [25], [26]. Similar analyses for damaging borers and sap feeders may be appropriate, given their current and projected costs, and the need to optimize spending allocations given current declining budgets.

Our framework can incorporate new information as it becomes available, including explicit cost estimates for additional species and cost categories (Appendix S1). For instance, by causing tree mortality, defoliation and reduced growth of their hosts, non-native forest insects can have important direct and cascading effects on non-market ecosystem services such as water and air quality, nutrient cycling, climate regulation, disease control, and recreation and cultural services [20], [27], [28]. Furthermore, non-native forest pests threaten native species and entire ecosystems such as the Fraser fir forests of the southern U.S. Appalachian Mountains, the rare Carolina hemlock trees, and redbay trees in the southeastern coastal plains [29], [30], [31], [32]. As data become available for these types of damages caused by exemplary “poster” pests, our framework can be used to estimate guild-wide ecosystem services losses.

Our study provides the most comprehensive estimates of costs of forest invaders currently available for the United States, the probability of future costs and, therefore, the benefits of reducing the rate of invasion. We identify the insect guilds most responsible for, and the societal elements most affected by, these damages, and we provide insight into the introduction pathways that could be targeted by management actions. Our work can be used in quantitative cost-benefit analysis of the preventative measures that are widely regarded to be the best option for addressing invasive species [13], [33]. For example, targeted import taxes or fees have been proposed as a means of generating funds to pay for practices to reduce introductions or to eradicate or control invasive pests that have already established [34]. Development, implementation and justification of such policies will require these estimates of nationwide economic damages and the sectors affected by invasive pests. Our analytical framework can be used in any country where data are available and can be easily adapted for estimating costs in a variety of natural resource sectors in addition to invasive species, including fire, disease, and water quality, at scales from municipalities to nations.

## Supporting Information

Appendix S1 **Detailed economic and modeling methods.** Part I describes the economic methods and data sources. Part II describes the database of non-native forest pests and the classification of their impacts. Part III describes the Bayesian model used to estimate total costs.
(DOC)

Figure S1 **Study area of U.S. counties infested at the beginning of the study period (shaded green) and at the end of the study period (shaded yellow) by the emerald ash borer (top), the gypsy moth (middle), and the hemlock wooly adelgid (bottom).**
(TIF)

Figure S2 **Sensitivity of total cost estimate to the lower threshold for each guild and cost category combination.** Low and high represent posterior values obtained using a lower threshold one order of magnitude below and above (respectively) expert opinion (medium). Mean and 90% Bayesian credible intervals illustrated.
(TIFF)

Figure S3 **Sensitivity to the lower threshold of the probability of a new pest as damaging or more damaging than the poster pest for each guild and cost category combination.** Low and high represent posterior values obtained using a lower threshold one order of magnitude below and above (respectively) expert opinion (medium). Mean and 90% Bayesian credible intervals illustrated for each guild and cost category combination.
(TIFF)

Table S1 **Ten-year time horizon for calculating poster pest damages.**
(DOC)

Table S2 **Host tree density on developed land for the study areas corresponding to the emerald ash borer and hemlock woolly adelgid.**
(DOC)

Table S3 **Ash density by land use and diameter class for the city of Chicago.**
(DOC)

Table S4 **Management costs for homeowners and community managers.**
(DOC)

Table S5 **Parameters for the model of timber losses to forest landowners.**
(DOC)

Table S6 **Lower economic threshold of damages by damage category.**
(DOC)
