# Supplementary material for: Economic Impacts of Non-Native Forest Insects in the Continental United States
Source: PLoS One. 2011 Sep 9;6(9):e24587. doi: 10.1371/journal.pone.0024587 (PMC3170362; doi:10.1371/journal.pone.0024587)
Supplement: Appendix S1 — Detailed economic and modeling methods. Part I describes the economic methods and data sources. Part II describes the database of non-native forest pests and the classification of their impacts. Part III describes the Bayesian model used to estimate total costs. (DOC) [file pone.0024587.s001.doc]

Appendix S1

**Methods**

Our quantitative analysis of nation-wide economic impacts from non-native forest insects was based on three key elements: I) an economic assessment, by cost category, of one poster pest for each of three insect guilds, II) a database of all non-indigenous forest insects established in the US by feeding guild and the separation of these species into three damage categories, and III) an integrative model which used these data to estimate cost probability functions for each guild and cost category.

I. Economic Costs and Losses from Three Poster Pests in the United States

Poster pests represent the most economically damaging non-native forest insects in each of three feeding guilds: phloem and wood borers (emerald ash borer, *Agrilus planipennis*), sap feeders (hemlock woolly adelgid, *Adelges tsugae*), and foliage feeders (gypsy moth, *Lymantria dispar*). We estimated economic damages associated with each poster pest for three sectors of the forest economy (government, household, and market) in five cost categories: federal government expenditures, local government expenditures, household expenditures, residential property value losses, and forest landowner timber losses. These five cost categories represent measurable and substantive direct costs and losses caused by non-native forest insect pests. Although other sectors may be negatively affected by these pests, the costs are not as great (e.g. nurseries) or not readily measureable with available data and models (e.g. timber mills and ecosystem services). Input-output models of economic activity fail to account for substitutability by consumers and businesses and do not offer a defensible lower bound for damages. Other models such as those for measuring ecosystem services losses have not been used for small (in the sense of a single tree species dying as opposed to an entirely new land cover such as agriculture) environmental changes at a broad geographic scale.

Federal government expenditures are incurred by federal agencies for regulatory activities, survey and detection, outreach and education, biological control, methods development, and research. Both local government and household expenditures include the removal and treatment of trees affected by an invading insect. Because some of the expenditures of the federal government may include transfers to local government, we note that estimates of federal government expenditures should not be added to local government expenditures, as this would constitute double counting. Government and household expenditures are economic transfers because they represent an exchange of currency from one governmental or private entity to another. There is an opportunity cost to these transactions in which some sectors of the economy lose wealth (such as homeowners) while other sectors gain (such as tree removal firms). Since the transfers occur in response to an unwanted insect infestation, there is likely a preferable alternative for the expenditures, by the government and household. Thus, the transfer will lower economic welfare because choosing to spend on the insect infestation is not the choice that has the lowest opportunity cost. A general equilibrium model, outside the scope of this analysis, could capture the loss of economic welfare from these transfers.

In addition to economic transfers, non-native forest insects cause a loss of economic value due to the loss of aesthetics, ecosystem services, and commerce. The loss of aesthetic and ecosystem service value to the household sector can be measured using the hedonic property value method. We used existing microeconometric estimates of the contributions made by trees to residential property values [1,2,3] in combination with models that estimate the spread of economic damage, to estimate broad-scale economic impacts on residential property owners due to the loss of services supplied by residential trees. Although the loss of residential property value is transitory, as trees may be planted or naturally regenerated, the combination of slow biological growth and positive discount rates suggests that the loss of mature trees to non-native forest pests causes substantial losses in value for current homeowners. Because some of the value loss induces homeowner expenditures for replacement trees, we note that estimates of residential property value losses should not be added to homeowner expenditures as this would constitute double counting.

Commercial losses are represented by the loss of timber value to forest owners. Decreased timber growth or increased timber mortality may be offset to a considerable degree by compensatory growth of remaining healthy trees and this should be accounted for. As computed here, timber losses do not include expenditures by landowners to protect their forest from insects, or the possibility of preemptive or salvage logging which would mitigate timber losses to forest owners. Further, it is assumed that timber buyers can perfectly substitute other species for the timber volume lost to non-native forest pests.

We calculated economic damages for each poster pest and cost category for ten-year horizons. We chose a ten-year horizon to represent a decade of average damages from the poster pest and for which the best data on poster pest damages were available (see below). In order to meet these criteria, the ten-year horizon differs across poster pests. Due to data limitations, the time horizon of the federal expenditures damage category is different from the time horizon of the other damage categories for the same poster pest. This is acceptable because damage categories are never summed. The present value of a stream of damages that extend beyond the ten-year horizon, e.g. lower forest landowner timber revenues for thirty years, because of mortality of a forest tree species that occurs within the ten-year horizon is included in the tally of damages for that ten-year horizon.

We present the methods to model economic damages for each poster pest and cost category in three main sections: A) Estimation of Infestation and Host Mortality, B) Economic Methods, and C) Review of Economic Model Assumptions. Each of the first two main sections has a few subsections. The subsections to the Estimation of Infestation and Host Mortality are 1) the study area and time horizon, 2) estimation of host tree numbers in developed areas, and 3) prediction of infestation and mortality. The subsections to the Economic Methods are a) federal government expenditures, 1) local government and homeowner expenditures, 2) household property value losses, 3) forest landowner timber value losses, and 4) ecosystem services losses. The last main section reviews the assumptions of the economic model and how uncertainty could affect economic damages.

# A. Estimation of Infestation and Host Mortality

The initial phase of the analysis was to estimate the extent of infestation and host mortality associated with each poster pest over a ten-year horizon. We accomplished this in three steps: (1) we specified the study area and ten-year horizon; (2) we estimated the number of host trees on developed land within communities within the study area; and (3) we predicted the counties within the study area that will become infested, and the number of trees that will be affected, over the selected 10-yr horizon.

*1. Study area and time horizon*

We specified the time horizon and associated study area for each poster pest based on two criteria i) availability of accurate and reliable data, and ii) average damages for the poster pests. The average damages do not occur just after the pest is established because the range and density of the infestation is small, and likewise the average damages would not occur once most of the host species have already been killed. Average damages are likely to occur in a time frame between the establishment and the complete expansion of the pest.

Emerald ash borer (EAB), Agrilus planipennis Fairmaire (Coleoptera: Buprestidae), an exotic beetle native to Asia, was identified as the cause of widespread ash mortality discovered in southeastern Michigan in July 2002 [4]. Injury caused by larvae feeding in the phloem (inner bark) and along the outer surface of sapwood disrupts the ability of trees to transport water and nutrients. In its native range, this insect functions as a secondary pest agent, colonizing stressed and dying trees [5]. Ash species in North America, however, lack any coevolutionary history with EAB and healthy, as well as stressed trees, are colonized and killed. Emerald ash borer likely arrived in the United States on wood packaging material used for cargo originating in Asia [4,6]. Although adult beetles are active fliers, long range dispersal has resulted from the inadvertent human transport of infested ash trees, logs or firewood. As of June 2010, EAB populations had been found in 14 states and two Canadian provinces [7,8]. While EAB host preference or host resistance varies among North American ash species, all native ash species are likely to be susceptible, especially when EAB densities are high [6,9]. The expansion of the range infested by EAB (Figure S1) [10] and the anticipated economic costs likely to be incurred were estimated for the period of 2009 to 2018 for all damage categories except federal expenditures (Table S1). The period of 2009 to 2018 is more representative of a decade of average damages for EAB than 2002 to 2009 because the borer was just identified and beginning to spread in 2002, so the damages are lower than the average. Because federal expenditures for EAB were available beginning in 2003, and we preferred existing expenditure data when available, the period of 2003 to 2012, with forecasts for 2009 to 2012, was used for calculating the federal expenditures on EAB (P. Chaloux, personal communication).

Hemlock woolly adelgid (HWA), Adelges tsugae Annand (Hemiptera: Adelgidae), is an exotic, sap-feeding insect discovered in central Virginia in 1951. Eastern and Carolina hemlock have little resistance to HWA and are often damaged and killed within a few years following adelgid establishment [11]. Scientists recently determined that HWA populations established in the eastern United States originated from southern Japan [12]. HWA has now infested hemlock stands from northeastern Georgia to southeastern Maine and as far west as eastern Kentucky and Tennessee [13]. In the ten years from 1998 to 2007, HWA infestation and damages accelerated because the pest reached high levels, especially in the southeast U.S. [14]. Research efforts were expanded beginning in 1998, providing data related to HWA biology and impacts. This period represents average damages for HWA because the insect’s range had expanded outside New England but had not yet completely infested the southern states [13]. There may be cycles in the intensity of adelgid activity, but this has not been documented at a regional level. The study area associated with this study period is shown in Figure S1 [15]. Because federal expenditures for HWA are available beginning in 2000 and existing expenditure data are preferred, the slightly later period of 2000 to 2009 is used for calculating the federal expenditures on hemlock wooly adelgid (N. Schneeberger, R. Weeks, personal communication).

The gypsy moth (GM), *Lymantria dispar* Linnaeus (Lepidoptera: Noctuidae), originally evolved in Europe and Asia and was accidentally introduced to Massachusetts in 1868. About twelve years following introduction, the first outbreaks began and despite attempts to eradicate the gypsy moth, its range has continued to expand [16,17]. In the years 1988 to 1997, the gypsy moth caused heavy defoliation throughout its expanding range, which extended from Maine to southeastern Virginia and as far west as Michigan’s Upper Peninsula shown in Figure S1 [18]. The period of 1988 -1997 represents approximately average damages caused by gypsy moth; this period does not include the largest regional outbreak (early 1980's) but it does include the second largest outbreak (early 1990's). Also, during this period, the range had expanded outside of New England and it was repeatedly eradicated from western states [19]. Detailed gypsy moth data have been available since the early 1980s. Federal expenditures for GM are more readily available beginning in 1998, so the period of 1998 to 2007 is used for calculating federal expenditures on gypsy moth (W. Fussell, personal communication). Note that this time frame includes federal expenditures for the “slow the spread” program [20]. This makes federal expenditures significantly larger than the federal expenditures during the 1988 to 1997 time horizon, but still representative of average expenditures because the “slow the spread” program has been fully implemented since 1999.

We summarize the time periods for calculating damages for the poster pest damage categories in Table S1.

*2. Estimating host tree numbers in developed areas*

We estimated the number of host trees on developed land in U.S. Census-defined communities using a digital map of communities developed by the U.S. Forest Service from the 2000 U.S. Census [21]. Communities are defined as places of established human settlement and may include both developed and undeveloped land within their boundaries. We use communities and not urban areas as geographic units in this study because communities have geopolitical boundaries and people within these jurisdictions are likely to organize and manage their trees in response to infestation as a group. Host trees on developed land within communities represent trees with the highest priority to treat or remove. We excluded all trees in naturally regenerating forests from this component of the analysis. We identified developed land using the 2001 National Land Cover Database [22], which is a land cover classification system derived from satellite imagery and consistently applied with a 30 × 30 m^2^ resolution over the United States [23]. The NLCD 2001 has four developed land cover classes based on the percentage of impervious surface and vegetation cover [24].

We estimated the numbers of trees on developed land in communities using tree inventories for cities (usually single communities), and regions (which may include several communities) obtained from web sites, publications, and personal communication with city foresters [25,26]. Average densities of ash and hemlock trees on developed land within the study areas of the emerald ash borer and hemlock woolly adelgid, respectively, are listed in Table S2. We do not estimate numbers of trees for gypsy moth because, rather than one species or one genus, many tree species are susceptible to the moth and because area damaged (defoliation) is exhaustively mapped each year and available as GIS data. The calculation of expenditures for gypsy moth was derived by combining these data on the area affected by the moth with the per acre expenditures directed at it. Average host tree density for each study area is calculated as the simple average of host tree densities in cities and regions within the study area and with available tree inventory information. The inventories included estimates of the total number of host trees within city or regional boundaries, including trees on public and private lands. For each city or region, we divided the number of host trees by the area of canopy cover on developed land to obtain an estimate of host tree density.

The tree inventories for cities and regions provided the basis for estimating the number of host trees on developed land within communities. First, we divided the study area into mapping zones. The mapping zones are from the NLCD 2001 and represent areas of relatively homogenous landform, soil, vegetation and spectral reflectance [24]. Then, we assigned each city or region to a mapping zone and computed average tree host density (trees per ha cover) for the zone. For example, if forest inventory information is available for three cities in a mapping zone, the average host density of the three cities is the host density for the zone. Finally, we multiplied the average host tree density by the area of tree cover on developed lands in communities to estimate number of host trees in the communities. If inventory data for a particular zone were unavailable, we substituted host tree density from the nearest zone.

Recognizing that the cost of managing host trees in infested areas is a function of tree size and land use, we also estimated the number of host trees by size class and land use in the developed portion of communities in each mapping zone. The extensive ash tree inventory for the city of Chicago includes estimates of all trees within the urban boundary for seven land uses and several diameter classes [25]. From this detailed inventory (Table S3), we computed host tree density (trees per ha cover) for residential areas (single-family, multi-unit, and planned development), non-residential areas (downtown, industrial, open space, and commercial) and three tree diameter classes (0-30 cm, 30-61 cm, and > 61 cm) [25]. We apply the distribution of ash densities from Chicago of the seven land uses and three diameter classes to all the cities and regions in Table S2. Street trees are the responsibility of the community – consequently, we included street trees in the category of non-residential areas. We reallocated 10% of trees from residential areas to the non-residential areas to account for the street trees, based on estimates of the number of street trees in Indianapolis [27]. Because most city tree inventories only include estimates of numbers of host trees and no information about land use, we use the relative host tree densities across land use and size classes in Chicago to extrapolate host tree densities by land use and size class in each of the other cities.

To account for development outside communities, we expanded the land base to include all developed land as defined by the NLCD 2001. We did this with the contiguous land base of the counties, rather than the communities, of the study area. We estimate the number of host trees on developed land both inside and outside communities. The proportions of trees in residential and non-residential areas are assumed to be the same as for the city of Chicago because we do not have further information on the breakdown of trees by land use.

*3. Predicting infestation and mortality*

We used spatio-temporal dynamic models for EAB and HWA to predict their spread across the relevant study areas. Historical spatio-temporal data were available for GM, so modeling was not required.

We used a probabilistic model of EAB spread to compute 100 scenarios of EAB establishment across the study area over the next decade, 2009-2019 [10]. The model uses a negative exponential function to predict the annual probability that EAB in an infested cell will spread and establish a population in a vacant cell at a detectable level. The probability of spread, *p,* depends on the distance, *d* (km), between cell midpoints:

$p=0.94e^{-0.06d}$ (1)

In each scenario, the model begins with the locations of known EAB infestations in the U.S. and Canada in March 2009 and predicts the spread of the infestations for 10 years to March 2019. The two parameters of the probability model were selected by contrasting the predictions from simulations starting with a single infestation near the initial infestation in Wayne County, Michigan, in 1994 [28] to the observed infestations as of March 2009.

We overlaid a map of counties on the center points of the grid to predict whether each county would be infested each year. A county was considered infested when EAB is detected in at least one grid point within the county. Once an infestation has been detected in a county, it takes time for EAB to spread and infest all of the ash trees in the county. Smitley et al. [29] estimated that ash decline moved outward from a point of infestation at a rate of 10.6 km per year. Evidence from U.S. Forest Service Forest Inventory and Analysis plots suggests that catastrophic ash mortality in a county becomes apparent about five years after an infestation has been detected there [30]. From this evidence, we assumed that the percent of the ash that is infested in a county increases linearly from 0-100% in five years following the detection of the initial infestation.

We used a time series model to statistically estimate the spread of HWA induced severe hemlock defoliation in New Jersey residential forests, and we applied this model to the HWA study area. We obtained geo-referenced time series data for counties with a hemlock woolly adelgid infestation in New Jersey for the years 1992-2001 [31] and intersected them with geo-referenced data on residential neighborhoods. Holmes et al. (2010) [32] fit a quantile regression model to predict the effective range radius of the area of severe hemlock defoliation in New Jersey residential forests, ERR_t_ (km/yr), as a function of elapsed time since first infestation (E_t_) and hemlock basal area (HBA_t_):

ERR_t_ = 0.05E_t_ + 1.01HBA_t_ (2)

Dividing the predicted hectares of residential severe hemlock defoliation by estimates of total residential hemlock area in New Jersey yields an estimate of severe defoliation over a ten-year period of observation. Summed over the ten-year period, the cumulative residential severe hemlock defoliation in New Jersey was roughly 25% of the area in residential hemlock stands. Based on consultation with supervising foresters of Rhode Island, Massachusetts, and New Hampshire (B. Payton, personal communication), the adelgid population builds slowly from the initial infestation before damages become apparent. Hemlock mortality in a county increases linearly by 1.0%, beginning ten-years after the initial detection. The counties in New Jersey were infested more than ten years before mortality was observed. Data on initial detection were provided by the Forest Health Protection unit of the US Forest Service [15].

The historical spatio-temporal spread of gypsy moth is well documented and predictive models were not required. Rather, we determined the percentage of forest defoliated by gypsy moth from 1988-1997 by county from annual aerial sketch maps of defoliation across states in the generally infested zone for gypsy moth. These data were compiled by the Forest Health Protection unit of the US Forest Service [33].

### B. Economic Methods

Categories of economic damages from non-native forest insects include federal and local government expenditures, costs to homeowners, losses to homeowners measured by willingness to pay, and market losses to forest landowners such as timber. All economic damages are in 2009 U.S. dollars using the implicit price deflator for GDP.

*1. Federal government expenditures*

The analysis of federal expenditures relied upon historical and, in the case of EAB, anticipated expenditure records. Federal agencies with substantial expenditures targeted at EAB are the US Department of Agriculture Animal and Plant Health Inspection Service (APHIS), Forest Service (USFS), and the Agricultural Research Service (ARS). From 2003-2012, these agencies were estimated to spend the following amounts: 1) APHIS $313 million (P. Chaloux, personal communication), 2) USFS $26 million (N. Schneeberger, personal communication), (2) APHIS $313 million (P. Chaloux, personal communication), and 3) ARS $3 million (K. Hackett, personal communication). In total, we predict federal expenditures for the ten year period on EAB to be roughly $342 million.

Federal agencies with substantial expenditures targeted at HWA are the USFS and APHIS. From 2000-2009, these agencies were estimated to expend the following amounts: (1) USFS = $38 million, and (2) APHIS = $0.6 million. In total, federal expenditures for the ten year period on HWA is roughly $39 million (N. Schneeberger, R. Weeks, personal communication).

Federal agencies with substantial expenditures targeted at gypsy moth are the USFS, APHIS, and the National Institute for Food and Agriculture (formerly the Cooperative State Research, Education, and Extension Service (CSREES)). From 1998-2007, these agencies were estimated to expend the following amounts: 1. USFS = $217 million (with $19 million on research and development, $48 million on suppression, and $150 million on the Slow the Spread program) (E. Sills, personal communication), 2. APHIS = $78 million (W. Fussell, personal communication), and 3. CSREES = $3.4 million (M. Purcell, personal communication). In total, federal expenditures for the ten year period on GM was roughly $298 million.

### *2. Local government and homeowner expenditures*

Expenditures by homeowners and local governments confronted with EAB and HWA are largely unknown and were therefore estimated using microeconomic models of optimizing behavior. Expenditures by homeowners and local governments confronted with GM were based on historical expenditure data.

The costs of removal and replacement depend on tree size (Table S4), with community managers paying slightly less than homeowners. Removal and replacement costs were computed using an online calculator [34] and were calibrated for trees of differing size (15 cm, 45 cm, and 76 cm diameter trees). We assumed insecticide treatment for EAB involves injecting a systemic pesticide directly into the base of a tree, and is required every two years [34,35]. Treatment for HWA was assumed to be a basal soil drench, active ingredient imidacloprid, which is required at a minimum of every two years [36, M. Raupp, personal communication].

We assumed that homeowners and local governments confronted with EAB or HWA maximize the present value of a stream of benefits and costs by choosing among four actions: 1) do nothing, 2) remove trees, 3) remove and replace trees, or 4) treat trees with a pesticide to prevent injury from the pest. The homeowner and tree manager models assume the insecticide is nearly 100% effective at preventing trees from being infested for the interval of effectiveness found in scientific studies [35,36]. The treatment was available to households and tree managers at the beginning of the study period for EAB in 2009 and for HWA in 1998 [35,37]. We formulate a discrete-time dynamic programming model to determine the optimal action as a function of tree size [10]. Based on this analysis, we imposed the following rules regarding treatment and removal for the homeowner and community manager models: 1. When a county becomes infested, all larger trees (> 45 cm diameter for homeowners and > 61cm diameter for tree managers) are treated with a preventative insecticide immediately and then biannually until the end of the 10-yr horizon, 2. Smaller trees (< 45 cm diameter for homeowners and < 61cm diameter for community managers) are removed and replaced at the time of infestation, and 3. Removal, replacement, and treatment occur at the rate of 20% of the ash trees each year following the county’s initial infestation [10], and 1% of the hemlock trees ten years after the county’s initial infestation (K. Lombard, B. Payton, personal communication). The decisions are optimal according to the model, but sentimental attachment, limited budgets, or uncertainty surrounding the effectiveness of the treatment, could result in a different decision by homeowner or community manager.

A sensitivity analysis considered the total expenditures under the assumption that all ash trees in communities are removed and replaced at once [10]. The total cost in this case was double the average estimate. Another sensitivity analysis considered damages when removal and replacement of ash trees occur on developed land outside of communities, and this nearly doubled the average estimate as well. The sensitivity analyses indicate that damage estimates could conceivably be double on the upper end in extreme cases. On the lower end, since damage estimates could conceivably be half but not zero, sensitivity analysis suggests the distribution around the damage estimates could be skewed upwards. While many environmental and economic factors influence potential damages, our estimates have to rely on the best available data.

The annual costs of treatment and removal and replacement were discounted to the present using a 2% real discount rate. Howarth [38] observes that the future benefits of a public good should be discounted at a rate close to the rate of return for risk-free financial assets, even when the public good has risk characteristics equivalent to those of risky forms of wealth.

The cost to households of a GM infestation includes suppression (e.g. ground spraying, banding, and scraping egg masses) and mitigation (e.g. fertilizing trees, treatment for gypsy moth rash, power-washing homes, additional car washes and maintenance of pools, and the removal and replacement of dead trees), which we estimated is on average $187 per household [43]. We estimated the annual number of affected houses in a county as the product of the annual percentage of a county defoliated and the number of potentially affected houses with one or two units in the county. The number of potentially affected houses was based on the NLCD classification of the area of tree canopy on developed land, the proportion of land that is residential, and the number of houses per hectare of developed land. The purpose of this approach was to estimate houses with tree canopy because only houses with trees would be affected by the moth.

To ensure that this approach did not overestimate the number of potentially affected houses, we capped the estimate by the number of housing structures with one or two units from the Census. The annual homeowner cost of GM was calculated as the product of the average cost per household of GM, and the annual number of affected houses, summed over all the counties in the study area. The annual local government expenditures on GM was the product of the average per hectare cost, which is the average per household cost multiplied by twelve, for the number of homes per hectare that corresponds to the median lot size in the U.S., and the annual number of affected hectares for all the counties in the study area. The estimate of the average per hectare cost of gypsy moth to the local government is based on the assumption that there are twelve homes per hectare. The local government likely has lower suppression and mitigation costs than households because of economies of scale, but the concentration of damages on non-residential land is greater since this includes forested open space.

*3. Household property value loss*

We calculated homeowner losses associated with damage from EAB and HWA by calculating the sum of i) the net present value of the reduction in property value caused by having a dead tree on the property and ii) the net present value of the reduction in property value caused by a reduced tree canopy [39].

$HomeownerLoss=\theta\cdot P\cdot H\left(DV_{1}\cdot\left(\frac{1}{r}\left(1-\frac{1}{\left(1+r\right)^{T_{1}}}\right)\right)+DV_{2}\cdot\left(\frac{1}{r}\left(1-\frac{1}{\left(1+r\right)^{T_{2}}}\right)\right)\right)$ (3)

where $\text{θ}$ is the proportion of homes affected by the insect infection, r is the real rate of discount, P is the median value of a single-family home, H is the number of potentially affected houses, $\text{DV}_{1}$ is the percentage discount to home price of having a dead tree on a property, $\text{T}_{1}$ is number of years a dying or dead tree is on the property, $\text{DV}_{2}$ is the percentage discount to home price of having reduced tree canopy on the property, and $\text{T}_{2}$ is the number of years the property has reduced tree canopy.

The proportion of homes affected is the product of the proportion of the affected trees and the proportion of homes with host trees. The proportion of affected trees is based on the spread model, whether the insect infestation has reached the homes, the rate of insect damage, and how quickly host trees die once the insect infestation arrives. The proportion of homes with host trees is based on the number of host trees per home (which comes from the earlier estimate of the number of host trees) and the number of homes on developed land with canopy cover.

The median value of the single family home, which depends on the county where the home is, comes from the Census. The home value, which is the average for the period 2005 to 2007, is converted to 2009 dollars. The number of potentially affected houses was based on the NLCD classification of the area of tree canopy on developed land in communities, the proportion of land that is residential, and the number of houses per hectare of developed land. The purpose of this approach was to estimate houses with tree canopy because only those houses with trees on the property would be affected by one of these poster pests. To make sure this approach did not overestimate the number of potentially affected houses, we cap by the number of housing structures from the Census. This approach assumes homes have a 100% tree canopy, which potentially underestimates the number of affected homes because often homes have only partial tree canopy.

Several studies suggest a discount from having a dead and dying tree close to a property, which is approximately 5% [1,2,3], and this lasts for roughly three years until the removal of the dead tree makes property values return close to normal [3]. For EAB, where numerous trees may be dying on a residential property, 5% may be an underestimate of the discount. Several studies suggest a premium from having tree canopy near a property, which is around 0.5% [40,41,42]. We account for the loss of value because a healthy tree is gone with the 0.5%. In the case of insect infestation and the removal of dead trees, this implies diminished tree canopy and a discount of 0.5% for roughly ten years before a replacement tree grows sufficiently to compensate property values.

For HWA, property value loss for exurban households is available from statistical estimation of a spatial-dynamic value transfer model using spatially referenced data of hemlock forests and the NLCD [32,42]. Recognizing that economic impacts of biological invasions occur where biological processes intersect the economic landscape, Holmes et al. [32] define the area of economic damage (AED) as the sum of all areas on the physical landscape that sustain economic damage from a biological invasion.

We computed losses to homeowners from GM differently because GM causes defoliation of many species of host trees rather than mortality of a single species of host tree. The willingness to pay to avoid gypsy moth damage includes the loss of value from nuisance, defoliation, and mortality, which we estimate is on average $503 per urban household and $433 per rural household [43]. We include this willingness to pay loss in the property value loss category because the two methods measure roughly the same values, in the case of trees on a homeowner’s property, where we would expect there to be minimal non-use value. The willingness to pay method captures non-use values, but trees on a homeowner’s property are usually not unique, suggesting non-use values are not a large component of the total value.

The annual number of affected households in a county was based on the product of the annual percentage of a county defoliated and the number of housing structures with one or two units in the county. To distinguish whether the counties were urban or rural, we used the 2003 Economic Research Service Rural-Urban Continuum Code. We calculated the annual homeowner willingness to pay to avoid gypsy moth as the product of the average per household willingness to pay to avoid gypsy moth and the annual number of affected households for all the counties in the study area.

Homeowner expenditures are a component of homeowner losses, so the two categories should not be added to avoid double-counting. Further, the different methods for calculating homeowner expenditures and losses mean that expenditures may not be nested seamlessly within the losses. Expenditures do not rely on estimation of the prices, since tree removal and treatment costs are available from the market, and thus are less susceptible to statistical error than the estimation of the losses. Losses are important to calculate because they are potentially much larger than the mere expenditures from the insect infestations. Calculating the homeowner losses with the hedonic property value method is possible because we assume the housing market operates similarly across cities in the United States. Localized changes in the health of host species affect a relatively small number of properties within communities, and the hedonic price function for each housing market remains unaffected. Under these conditions, the first stage hedonic price function can be used to compute losses in economic welfare for all property owners impacted by the changed conditions [44]. The loss of wealth from tree mortality occurs regardless of whether an impacted home is sold or not. Holmes et al. [32] show that a loss of tree health induces spillover losses from properties with impacted trees onto neighboring properties. In this study, we take a conservative stance and do not account for the loss of wealth on neighboring properties with non-impacted trees.

*4. Forest Landowner Timber Value Loss*

We calculated stumpage value losses from EAB, HWA, and GM based on the following assumptions: (1) timber mortality from the poster pests reduces timber harvest volumes for the impacted species (ash, hemlock, and oak) by the average volume harvested for each species within each impacted county; (2) reductions in timber harvest from poster pests are a small percentage of total harvest volume and can be fully offset by timber harvests of non-impacted timber species; (3) after 10 years, compensatory growth on non-impacted healthy trees eliminates all lost timber harvest volumes. Given these assumptions, timber prices within impacted areas do not change and the only timber market participants that (temporarily) experience economic loss are forest landowners with impacted stands. In the case where insect infestations are severe enough to shift the short-run stumpage supply curve due to salvage or pre-emptive harvesting, timber growers with non-impacted stands and timber buyers may be impacted via market price impacts [45]. In addition, catastrophic losses of timber inventory due to pests, diseases, or storms can cause longer-term impacts on stumpage supply due to relative scarcity [46]. The stumpage value loss to this group is calculated as the net present value of annual timber harvest value lost for the number of years (T) required for the forest to fully recover timber volumes:

$StumpageValueLoss=P\cdot HL\cdot\left(\frac{1}{r}\left(1-\frac{1}{\left(1+r\right)^{T}}\right)\right)$ (4)

where P is the weighted price of a cubic foot of timber (weights are volume-adjusted stumpage value of saw timber, pulpwood, and fuel wood for each impacted county), and HL is the annual volume of timber that would have been harvested had it not been killed by the insect infestation.

Timber harvest loss (in cubic feet) was computed for three forest products (saw timber, pulpwood, and fuel wood) for each impacted county. We averaged annual harvest of impacted timber species, by county, for the years 2004 to 2006. Harvest volume data for impacted species were provided by the US Forest Service Forest Inventory and Analysis Mapmaker [47]. Estimates of the proportion of harvest volume for each forest product class, within impacted species, were based on recent government harvest reports [48,49,50]. Stumpage prices were the average of 2008 saw timber, pulpwood, and fuel wood stumpage prices for the study area and come from recent government timber price reports [51].

We computed timber volume (in cubic feet) loss for three forest products: saw timber, pulpwood, and fuel wood. We averaged annual harvest by county for the years 2004 to 2006. Data were provided by the US Forest Service Forest Inventory and Analysis Mapmaker [47]. The proportion of harvest volume sold for the three forest products depends on the tree species harvested and was based on recent government harvest reports [48,49,50]. The price of a cubic foot of timber was the weighted stumpage value of the three forest products. Stumpage prices were the average of 2008 saw timber, pulpwood, and fuel wood stumpage prices for the study area and come from recent government timber price reports.

Following an insect infestation, the growing stock volume can increase substantially because of volume gains in unaffected tree species and even gains in affected tree species that benefit from a thinning of the weaker trees [52]. We assumed that impacted stands would fully recover due to compensatory growth, over a period of ten years. For the first two years, timber is salvageable because the host trees killed by the insect maintain commercial value. However, for the eight remaining years the timber value is completely lost because trees are no longer salvageable. Salvageable timber loses roughly twenty percent of its marketable stem, and thus we estimate loses twenty percent of its value.

Davidson *et al.* [53] and Morin *et al.* [54] estimated mortality of oak stands from ten to twenty percent after one defoliation from GM. However, compensatory growth may offset these initial losses [52]. Two consecutive heavy defoliations can cause mortality to reach twenty to fifty percent [53]. Consequently, we assume a twenty percent loss of timber harvest of the oak stands. In the rare event of three consecutive heavy defoliations, the mortality results in an additional ten percent loss of timber harvest. We summarize the parameters for the model of stumpage value losses in Table S5.

We note that the methods used here differ from the approach used by Pimentel *et al.* [55] in three important aspects. First, in Pimentel *et al.* [55], timber damages occur in the forest and economic impacts are measured in final product markets, in terms of a reduction in gross domestic product of timber-related industries. This approach implicitly assumes that timber buyers and wood products manufacturers will not substitute alternate sources of wood supply for insect-killed timber. In contrast, our model assumes that timber buyers perfectly substitute alternative supply sources. Only fifteen percent of the counties in the study area harvest ash and only three percent harvest hemlock; for the counties that do harvest ash and hemlock, this ash and hemlock harvest is only twenty and thirteen percent of the total harvest, respectively. Although more than eighty-five percent of the study area counties harvest oak, less than three percent of oak forests are severely defoliated by gypsy moth. Since only a small proportion of total harvest is affected by these insect infestations, industry should be able to readily find reasonably priced substitute timber. This assumption may not hold for specialty markets such as the use of white ash for the Louisville Slugger baseball bat, and there is still the possibility that an insect infestation could arise that threatens a dominant hardwood or softwood timber species. Second, Pimentel *et al.* [55] implicitly assume that all non-native insect pests have an equivalent impact on timber mortality. Our model assumes that poster pests have the greatest measurable impact, while the majority of non-native insects are innocuous or have moderate impact. Third, Pimentel *et al.* [55] implicitly assume that economic losses accrue to the entire timber inventory killed by non-native forest insects. However, much of the timber inventory is not subject to harvest (e.g., the timber is inaccessible or the landowner has other objectives). In our study, timber losses only accrue to that portion of the inventory that would otherwise have been harvested.

*5. Ecosystem services losses*

Healthy forests on public lands undoubtedly provide valuable ecosystem services. As trees die from insect infestation, there are direct and indirect impacts to wildlife, hydrology, soil erosion, carbon sequestration and other fundamental ecosystem functions and services, with many of these impacts interacting in complex ways [56]. To our knowledge no single study comprehensively addresses all ecosystem impacts for even a single pest, or aggregates local findings to the full range of the host species. An adequate willingness to pay study would have to be carefully tailored to the specific ecosystem services trees provide and would have to be framed at the continental scale of the infestations by the poster pests. To our knowledge, no study like this has been conducted.

The limited research of losses on public lands focuses predominantly on recreation but may also include other ecosystem services. Two studies identify household willingness to pay to protect forests on public lands from widespread damage of insect infestation. Walsh *et al.* [57] find an annual willingness to pay of $47 per household to protect five million hectares of mixed-age ponderosa pine in the Rocky Mountains from the mountain pine beetle. Kramer *et al.* [58] find an annual willingness to pay of $28 per household to protect twenty-seven thousand hectares of high elevation spruce-fir forest in the southern Appalachian from exotic insects. No willingness to pay study exists to our knowledge that examines willingness to pay to protect urban forests on public lands.

An effective transfer of the household willingness to pay from the two available studies to the poster pest study area involves carefully matching the scope of the transfer studies to the scope of the policy sites. Two studies are relevant for examining the effect of the scope of the policy site on household willingness to pay. Loomis *et al.* [59] find that annual willingness to pay per household for forest protection from logging in Australia is $57 for six thousand hectares, $103 for seventy thousand hectares, and $100 for one hundred and twenty thousand hectares. Rollins and Lyke [60] find that annual willingness to pay per household for the creation of remote wilderness parks is $105 for one park, $162 for two parks, $191 for four parks, and $188 for ten parks. This suggests substantial diminishing household willingness to pay for greater scope. The willingness to pay studies on forest protection [57,58] and scope effects [59,60] are dated, and the scale of the insect infection is much smaller than the continental scale of the damages the poster pests cause.

C. Review of Economic Model Assumptions

An appropriate study area and time horizon of the poster pest infestations should balance the expansion of the range of the poster pest and the decline in the damages as areas already infested with the host species die back. Each time horizon chosen for the poster pests fits this definition. The number of trees in a developed area depends on the ash density estimates from a handful of urban forest inventories and the extent of the developed land area where trees will be treated or removed. Sensitivity of the extent of the developed land area where trees will be treated or removed finds that including developed land outside communities could double the damages [10]. Also, a sensitivity analysis for ash tree density suggests the damages could be half as small or half again as large [68]. The projected infestations by the emerald ash borer are largely insensitive to the spread model [10].

Local government and homeowner expenditures depend on per tree treatment and removal costs, and sensitivity analysis of the per tree costs finds the damages could be half as small or half again as large [68]. Household property values depend on how long and how much an infected tree discounts property values, and both are influenced by how long the infection remains on the property. The speed with which a community responds to an insect invasion thus influences the extent of the property value losses. The community and homeowner choice of removal and treatment of trees depends on many qualitative elements such as sentimental attachment, limited budgets, or uncertainty surrounding the effectiveness of the treatment. One influence on the forest landowner timber value losses is the time frame for compensatory growth to recover timber volumes. The benefits of the compensatory growth will depend on whether other valuable timber species are near the affected trees.

II. Database of non-indigenous forest insects

We assembled a list of non-indigenous forest insects with at least one recorded location of establishment in the continental United States [61].  We based our list on published lists and reports and communications with experts [62,63]. Species included in our list had to feed on at least one tree species found in native forest or common in urban forest settings (e.g. eucalyptus). We excluded species that have gradually expanded their range in a continuous fashion as a result of migration or climate change, and indigenous invaders (i.e. species native to the US introduced into previously uncolonized regions of the US). We excluded insects and pathogens that primarily colonize agricultural commodities, herbaceous plants, dead or processed wood as well as those that are primarily detritivores, predatory, parasitic, pollinators, aquatic, or that feed only occasionally on trees.

We assigned insect species to feeding guilds based on their dominant or most damaging feeding mode. Foliage feeders included insects that feed externally or internally on foliage, including leaf and needle miners. A few insect species that feed on meristematic tissue in apical or lateral shoots were included in the foliage-feeding guild because the damage associated with these species (e.g. loss of leaf area, topkill or loss of apical dominance) is similar to that caused by foliage feeders. Phloem and wood borers included species that feed aboveground on phloem, cambium or wood. Sap feeders included gall-forming adelgids, as well as other insects such as scales, aphids, psyllids and whiteflies. Species that feed primarily on roots, seeds, cones or fruit were assigned to an ‘Other’ guild and were not included in the current analysis. Insects that feed on different plant tissues as immatures and adults were assigned to the feeding guild most associated with injury or damage. For example, emerald ash borer was assigned to the phloem and wood borers guild because phloem-feeding by larvae causes tree mortality, while foliage-feeding by adults causes negligible injury.

We identified a subset of insect species from the original list, which included species of regulatory significance and species reported to cause a significant (above background levels) impact to live trees in some area of North America [61]. The poster pests were the most damaging of this subset. Impacts included tree mortality (including seedlings), canopy thinning or dieback (branch death), growth loss, defoliation, decreased reproduction or regeneration (e.g. seed or cone damage) or other ecological or aesthetic effects in forest or urban trees. We excluded pests that only cause economic damage to commercial fruit orchards. At least one published report of damage was required before a species could be included in the intermediate impact list. Sources of information included scientific journals, regulatory reports and university extension publications.

The database of species, as well as additional details about species attributes, is available through the NCEAS data repository [61].

III. Integrative Model

A. Theory

In this section, we develop the theory underlying our approach for estimating the cost caused by groups of non-indigenous forest insect pests. We provide both the generalized concepts and equations to allow transferability to other groups of invaders and other systems, as well as to demonstrate its application given the data available for our system.

Unfortunately, it is not feasible to conduct a thorough economic or ecological analysis of all pests in a rigorous manner. Indeed, even obtaining an analysis of a single pest is a major undertaking (see part I above). Thus, we developed methods to use all of our knowledge, to the maximum extent possible. Our full knowledge is comprised of both logical deductions and available data. With respect to data, we have information on 1) the non-indigenous forest insect species discovered (described in part II above); 2) a list of which of these species are damaging (part II); and 3) for three of the most damaging pests, we were able to derive economic estimates of damages (part I).

To estimate the overall damage caused by insect pests, we would like to sum the costs across all species. While this is not possible directly, as costs have not been quantified for most species, we propose that there exists a frequency distribution where different pests cause different levels of damages (henceforth termed cost curve, Fig 1). If we can describe this cost curve, we can perform additional analyses, such as estimating the costs from a pest guild or probabilities of new poster pests (see below).

Characterizing this cost curve is a two step process: 1) we need to define the shape of the distribution; 2) we need to determine how well each curve fits the data available.

*Step 1: The shape of the distribution*

While we do not know the shape of the distribution per se, through logic, we can roughly define some of the characteristics. Because available evidence suggests that low impact species are much more common than marginally damaging ones, with only a few high impact invaders [61,64], the frequency distribution should be concave in most of its range. We assert that unintentionally introduced phytophagous insects on balance do not have positive effects (i.e., cost ≥ 0). Thus we can describe the possible curves with relatively few models.

We considered 39 families of distributions and reduced these to four alternative models – the gamma, Weibull, power function and log-normal distributions, which all had the appropriate theoretic properties [65]. The other distributions were eliminated for a number of reasons: they did not have the appropriate constraints (e.g., negative values were possible, such as the normal or Cauchy distributions), they were special cases of the families considered (e.g., the exponential, Rayleigh, or Pareto were special cases or slight modifications of the Weibull), or they had other undesirable theoretic properties (e.g., Chi-Squared and F-distributions were constrained to integer parameters and therefore were excluded).

Our four models were defined by:

weibull

$pdf=\frac{k}{\lambda}\left(\frac{x}{\lambda}\right)^{k-1}e^{-\left(\frac{x}{\lambda}\right)^{k}}$ (5)

gamma

$pdf=\frac{x^{k-1}e^{-x/\theta}}{\Gamma\left(k\right)\theta^{k}}$ (6)

Power function

$pdf=\frac{cx^{c-1}}{b^{c}}$ (7)

And log-normal

$pdf=\frac{1}{x\sqrt{2\pi\sigma^{2}}}e^{-\frac{(\ln x-\mu)^{2}}{2\sigma^{2}}}$ (8)

Where pdfs are the probability density functions that describe the relative frequency of costs, x is the cost of a pest, and the other coefficients are fitted parameters. Thus, each cost curve is defined by the distribution model and the parameters within the given model (equation 5-8).

*Step 2: Fitting the data*

As our second step, we needed to relate each curve to our data. We used Bayesian analysis as a method to characterize the degree of uncertainty, given the data, and to estimate the relative support for a parameter set within a model. In the simplest case, where we have cost estimates for all pests,

$P(\theta|c)\propto P(c|\theta)P(\theta)\propto\left[\prod_{m=1}^{M} f(c_{m}|\theta)\right]P(\theta)$ (9)

where *P*(*θ* | **c**) is the posterior probability that a given set of parameters (*θ*) is correct given the observed costs (**c**) for all pests. *P*( *θ* ) is the prior probability, the term in the brackets is the likelihood function; $f(c_{m}|\theta)$ is the probability density function (*pdf*) of obtaining a cost *c_m_* for each pest *m,* given parameter set *θ*, and *M* is the total number of data points. This is a general formulation and the form of the likelihood function and parameter set *θ* can be very flexible, and can be applied to different models (e.g., equation 5-8).

Equation 9 is provided for generality in the development of our approach. It is operational when there are cost estimates for all species. However, in many instances, such as for forest insect pests, we do not have explicit cost estimates for all species. In such cases, it may be feasible to categorize costs into low, intermediate, and high, as we did for forest pests (Part II, [61]. Next we used expert opinion to define the threshold between low and intermediate costs (Table S6). We used the cost of the poster pest to define high damages. In summary, 1) we partitioned cost curves into three sections: low, intermediate, and high cost; 2) we determined the probability of a pest falling into each section by integrating under the curve for that section (Fig 1A), for each set of parameter values within each model examined; 3) we determined the relative support for a given parameter value and model, given the observed frequencies of pests in each category using:

$P(\theta|d)\propto\left[\prod_{i=1}^{I} P(low|\theta)\cdot\prod_{j=1}^{J} P(\mathrm{int}ermediate|\theta)\cdot\prod_{k=1}^{K} P(high|\theta)\cdot\right]P(\theta)$ (10)

Where *P*(*θ* |*d*) is the posterior probability that a given set of parameters (*θ*) is correct given the data (d), d is defined by the frequency of low, intermediate and high damaging species observed, I is the frequency of low damage species, J is the frequency of intermediate pests, and K is the frequency of highly damaging pests. The probabilities (P) were determined by integrating under the curves (equations 5-8). More generally, we can maximally use available data by mixing equations 9 and 10:

$P(\theta|d)\propto\left[\prod_{i=1}^{I} P(low|\theta)\cdot\prod_{j=1}^{J} P(\mathrm{int}ermediate|\theta)\cdot\prod_{k=1}^{K} P(high|\theta)\cdot\prod_{m=1}^{M} f(c_{m}|\theta)\cdot\right]P(\theta)$ (11)

Where notations are as defined in equation 9 and 10. Here, for all M species where we have explicit cost estimates, we directly use the probability density function. For all other I, J, and K species, we extract the maximum amount of information from the data, by categorizing them and integrating under the appropriate portion of the curve as explained above. In this way, we can use all the information available: 1) all of the non-indigenous forest insect species discovered; 2) a list of which of these species is damaging above some threshold cost; and 3) economic estimates of damages caused by pests.

B. Analysis

*1. Estimating distributions*

Each curve we generated described the variability in costs across pests (equation 5-8). However, we wanted to convert the output of our model from statistical curves and parameter values into more accessible meaningful measures: total costs of insect pests and probabilities of introducing a new poster pest. Analyses were conducted separately for each economic cost category, to avoid double counting and to identify the distribution of impacts among categories; and for each guild to control for modes of damage and vectors of transport.

Generally, to calculate the total cost of insect pests within each guild-cost category combination, using the maximum amount of information available, we sum the cost of all M measured pests which have been explicitly measured, with the estimated cost distribution of all other pests. For all unmeasured pests, our maximum information is the damage category into which they fall (low, intermediate, high), and the expected cost within that category. In notation:

$T=\sum_{m=1}^{M} c_{m}+\sum_{i=1}^{I} \frac{\int_{c=0}^{LT} c\cdot pdf(c|\theta)d\theta}{\int_{c=0}^{LT} pdf(c|\theta)d\theta}+\sum_{i=1}^{J} \frac{\int_{c=LT}^{HT} c\cdot pdf(c|\theta)d\theta}{\int_{c=LT}^{HT} pdf(c|\theta)d\theta}+\sum_{k=1}^{K} \frac{\int_{c=HT}^{\infty} c\cdot pdf(c|\theta)d\theta}{\int_{c=HT}^{\infty} pdf(c|\theta)d\theta}$ (12)

Where T is the total cost of insect pests, M is the number of pests for which we have explicit economic estimates, and I, J, and K are the numbers of low, intermediate and high damaging pests observed, respectively. LT is the lower threshold, HT is the higher threshold, c is the cost, and is the parameter set underlying the cost-curve frequency distribution. Thus, $\frac{\int c\cdot pdf(c|\theta)d\theta}{\int pdf(c|\theta)d\theta}$ is simply the expected cost (*E(c)*) within a given range of the cost curve, for a given model and associated parameter values. We used MCMC to estimate the posterior distribution in order to incorporate uncertainty in the parameter values (*θ*).

To estimate the probability (*p*) that a pest would be as damaging as the poster pest, we integrated under the part of the cost curve that was equal to or more extreme than the “high-cost” threshold (defined above). This probability can be scaled by the rate of new arrivals to estimate the probability that at least one new pest will be as damaging as the poster pest:

$E=1-(1-p)^{(Y\cdot R)}$ (13)

Where *Y* is the time horizon in years, *R* is the rate of introduction of pests from the guild of interest. In our example, two pests per year have been introduced between 1980 and 2006 and approximately 56% of them were borers [61].

We used the Bayesian approaches to generate uncertainty estimates for the derived variables –total cost and probability of poster pests. Specifically, we used the Metropolis-Hastings algorithm [66] (and flat priors) to determine the posterior distributions, and the probabilities that each set of parameter values (each curve) was correct (equation 11). The Bayesian posterior distribution described the parameter uncertainty. To account for model uncertainty, we used Bayesian model averaging across our four models [67].

Finally, because we used expert opinion to define the threshold separating low from intermediate damaging pests (Table S6), we also examined the sensitivity of our analysis by varying this threshold across two orders of magnitude. Thus, for instance, government expenditure for a low-cost pest was modeled with a threshold of $150, $1,500 and $15,000 annual costs over a ten year time window (Figs. S2, S3). Our model was robust to misspecification of the lower threshold.  Varying the lower threshold up and down over two orders of magnitude resulted in total cost estimates which were 110 and 93% our largest cost category, respectively, of the mean estimate produced using a lower threshold derived from expert opinion. For the poster pest probability, the alternate predicted values ranged between 89 and 120%.

Thus, we explicitly considered uncertainty due to variability across pests, parameter uncertainty, model uncertainty and sensitivity analysis, so that we could make use of the data available and report the levels of uncertainty to the most realistic degree possible. We applied this procedure separately for each cost category and for each guild.

Estimates of impacts associated with each guild could potentially be inflated if some insect species feed on the same tree species. For example, two tree-killing beetle species capable of killing the same tree species could both occupy a given location; including costs associated with each beetle species killing the same tree(s) would amount to “double counting”.

While we recognize the potential for such inflation of cost estimates, there are several reasons why this form of double counting is likely to be negligible. First, only 33% of non-native forest insects established in the US are polyphagous while 49% are monophagous [61]. Presumably, there are very few monophagous species that share host tree species. For the ca. 30 high-impact insect species that are not monophagous, by chance there may be a few cases of host overlap among the ca. 450 host tree species [69] present in the continental US. However, the fraction of species that overlap in space and time are likely to be minimal. Such overlap would only be relevant if the range of two pest species overlapped during the same 10-year interval we used for estimating costs. Finally, if two species share the same host species and overlap in space and time, it will not necessarily result in double-counting of costs as long as impacts associated with pests are not sub-additive. Most forest pests affect trees by reducing tree vigor, and generally these impacts are considered to operate in a cumulative manner [70]; additivity of impacts may thus be more likely than subaditivity. For these reasons, any inflation of pest impact estimates caused by overlap in host species ranges should be negligible.

*2. Theoretic analysis*

We validated our approaches theoretically, using simulations to generate “true” parameter values and data based on those true parameter values; we determined how well we could recapture those parameter values using equations 9 to 11. We tested for consistent deviations from the true parameter values (bias) as well as magnitude of deviations (accuracy). Our approaches were able to consistently recapture the underlying parameters – there was no bias and accuracy increased with information [equation 4 (full cost information) > equation 6 (mixed information) > equation 5 (frequency bins only], as well as with number of pests included in the analysis (sample size).

We further validated our approach for estimating our derived quantities of interest (total cost, and probability of a new highly damaging pest). For total cost, we generated random costs of pests from known underlying cost curves, to simulate a highly stochastic process. We compared the sum of the simulated pests to our predictions based on the procedure described above (“Estimating distributions”). Similarly, we followed this procedure to confirm our ability to predict the probability of new poster pests occurring. Using this validation procedure, we were able to verify that our method provides unbiased estimates of these quantities and that these estimators exhibit desirable asymptotic properties (posterior variance decreases monotonically as sample size increases).

**References**

1. Holmes T, Murphy EB, K (2006) Exotic forest insects and residential property values. Agric Resource Econ Rev 35: 155-166.

2. Huggett R, Murphy E, Holmes TP (2008) Forest disturbance impacts on residential property values. In: Holmes T, Prestemon J, Abt K, editors. The economics of forest disturbances: wildfires, storms, and invasive species. Dordrecht, The Netherlands:: Springer-Verlag. pp. 209-228.

3. Kovacs K, T. Holmes, J. Englin, J. Alexander (2011) The dynamic response of housing values to a forest invasive disease: evidence from a sudden oak death Infestation. Environ Resource Econ. 49: 445-471

4. Cappaert D, McCullough D, Poland T, Siegert N (2005) Emerald ash borer in North America: A research and regulatory challenge. Am Entomol 51: 152-165.

5. Liu H, Bauer LS, Gao R, Zhao T, Petrice TR, et al. (2003) Exploratory survey for the emerald ash borer, *Agrilus planipennis* (Coleoptera: Buprestidae), and its natural enemies in China. Great Lakes Entomol 36: 191-204.

6. Poland TM, McCullough DG (2006) Emerald ash borer: Invasion of the urban forest and the threat to North America's ash resource. J Forestry 104: 118-124.

7. US Forest Service (2010) Map and State EAB Information (http://www.emeraldashborer.info/map.cfm, April 14, 2010).

8. Michigan Department of Natural Resources (2009) Emerald Ash Borer Preparedness Plan. Forest, Mineral and Fire Management, Michigan Department of Natural Resources. 37 p.

9. Anulewicz AC, McCullough DG, Cappaert DL, Poland TM (2008) Host range of the emerald ash borer (Agrilus planipennis Fairmaire) (Coleoptera: Buprestidae) in North America: results of multiple-choice field experiments. Env Entomol 37: 230-241.

10. Kovacs KF, Haight RF, McCullough DG, Mercader RJ, Siegert NW, et al. (2010) Cost of potential emerald ash borer damage in U.S. communities, 2009-2019. Ecol Econ 69: 569-578.

11. Orwig DA, Foster DR, Mausel DL (2002) Landscape patterns of hemlock decline in New England due to the introduced hemlock woolly adelgid. J Biogeogr 29: 1475-1487.

12. Havill N, Montgomery M, Yu G, Shiyake S, Caccone A (2006) Mitochondrial DNA from hemlock woolly adelgid (Hemiptera: Adelgidae) suggests cryptic speciation and pinpoints the source of the introduction to eastern North America. Ann Entomol Soc Am 99: 195-203.

13. Morin RS, Liebhold AM, Gottschalk KW (2009) Anisotropic spread of hemlock woolly adelgid in the eastern United States. Biol Invasions 11: 2341-2350.

14. Morin RS, Liebhold AM, Luzader ER, Lister AJ, Gottschalk KW, et al. (2005) Mapping host-species abundance of three major exotic forest pests. Res Pap NE-726 (US Department of Agriculture, Forest Service, Northeastern Research Station).

15. USDA Forest Service (2009) Northeastern area state & private forestry hemlock woolly adelgid infestations by state and county. (US Department of Agriculture, Forest Service, Forest Health Protection, http://na.fs.fed.us/fhp/hwa/infestations/infestations.shtm, May, 6, 2009)

16. Liebhold AM, Halverson JA, Elmes GA (1992) Gypsy Moth Invasion in North America: A Quantitative Analysis. J Biogeogr 19: 513-520.

17. Liebhold AM, Tobin PC (2006) Growth of newly established alien populations: comparison of North American gypsy moth colonies with invasion theory. Popul Ecol 48: 253-262.

18. USDA Forest Service (2009) Gypsy moth quarantine maps (USDA Forest Service, Northeastern Research Station, http://www.fs.fed.us/ne/morgantown/4557/gmoth/atlas/#spread).

19. Johnson DM, Liebhold AM, Bjornstad ON (2006) Geographical variation in the periodicity of gypsy moth outbreaks. Ecography 29: 367-374.

20. Sharov AA, Leonard D, Liebhold AM, Roberts EA, Dickerson W (2002) "Slow the Spread": A national program to contain the gypsy moth. J Forestry 100: 30-35.

21. Nowak DJ, Greenfield EJ (2008) Urban and community forests of New England: Connecticut, Maine, Massachusetts, New Hampshire, Rhode Island, Vermont. Newtown Square, PA: U.S Department of Agriculture, Forest Service, Northern Research Station. 62 p. p.

22. US EPA (2001) National Land Cover Data (U.S. EPA, http://www.epa.gov/mrlc/nlcd-2001.html).

23. Homer C, Dewitz J, Fry J, Coan M, Hossain N, et al. (2007) Completion of the 2001 national land cover database for the conterminous United States. Photogramm Eng Rem 73: 337-341.

24. Homer C, Huang CQ, Yang LM, Wylie B, Coan M (2004) Development of a 2001 national land-cover database for the United States. Photogramm Eng Rem 70: 829-840.

25. Nowak DJ, Noble MH, Sisinni SM, Dwyer JF (2001) People & trees - Assessing the US urban forest resource. J Forestry 99: 37-42.

26. Sydnor TD, M. Bumgardner, A. Todd (2007) The potential economic impacts of emerald ash borer (*Agrilus planipennis)* on Ohio, U.S. communities. Arboriculture Urban For 33: 48-54.

27. Peper PJ, McPherson EG, Simpson JR, Vargas KE, Xiao Q (2008) Municipal forest resource analysis. Indianapolis, Indiana: Center for Urban Forest Research, U.S. Department of Agriculture, Pacific Research Station, Tech Rep. for Indiana Parks and Recreation Department. 2 pp.

28. Siegert NW, McCullough DG, Liebhold AM, Telewski FW (2006) Spread and dispersal of emerald ash borer: A dendrochronological approach. In Proceedings of the Emerald Ash Borer Research and Technology Development Meeting. US Forest Service, Forest Health Technology Enterprise Team, Pittsburg, PA. pp. 10.

29. Smitley D, Davis T, Rebek E (2008) Progression of ash canopy thinning and dieback outward from the initial infestation of emerald ash borer (Coleoptera : Buprestidae) in Southeastern Michigan. J Econ Entomol 101: 1643-1650.

30. Pugh SA (2010) Michigan’s forest resources, 2008. Research Note NRS-50. Newtown Square, PA: U.S Forest Service, Northern Research Station. 4 pp.

31. Royle DD, Lathrop RG (1997) Monitoring hemlock forest health in New Jersey using Landsat TM data and change detection techniques. For Sci 43: 327-335.

32. Holmes T, Liebhold A, Kovacs K, Von Holle B (2010) A spatial-dynamic model of aggregate property value losses from a biological invasion. Ecol Econ 70: 86-95.

33. Liebhold AM, Gottschalk KW, Mason DA, Bush RR (1997) Forest susceptibility to the gypsy moth. J Forestry 95: 20-24.

34. Purdue University (2009) Emerald ash borer in Indiana. (Purdue University, http://www.entm.purdue.edu/EAB/).

35. Herms D, McCullough D, Smitley D, Sadof C, Williamson R, et al. (2009) Insecticide options for protecting ash trees from emerald ash borer. Wooster, Ohio: North Central IPM Center Bulletin, Ohio State University Extension. pp. 12

36. Cowles RS, Montgomery ME, Cheah CAS-J (2006) Activity and residues of imidacloprid applied to soil and tree trunks to control hemlock wooly adelgid (Hemiptera:Adlegidae) in forests. J Econ Entomol 99: 1258-1267.

37. Steward V, Horner TA (1994) Control of hemlock woolly adelgid using soil injections of systemic insecticides. J Arboriculture 20 287-288.

38. Howarth RB (2009) Discounting, uncertainty, and revealed time preference. Land Econ 85: 24-40.

39. Kovacs K, Vaclavik T, Haight RG, Pang A, Cunniffe NJ (2011) Predicting the economic costs and property value losses attributed to sudden oak death damage in California (2010-2020). J Environ Manage 92: 1292-1302.

40. Anderson LM, Cordell. HK (1985) Residential property values improve by landscaping with trees. South J Appl For 9: 162-166.

41. Netusil N, S. Chattopadhyay, K. Kovacs. (2010) Estimating the demand for tree canopy: A seond-state hedonic analysis in Portland, Oregon. Land Econ 86: 281.

42. Sander H, Polasky S, Haight R (2010) The value of urban tree cover: a hedonic property price model in Ramsey and Dakota Counties, Minnesota, USA. Ecol Econ 69: 1646-1656.

43. Jakus PM (1992) Valuing the Private and Public Dimensions of a Mixed Good: An Application to Pest Control. Raleigh, NC: North Carolina State University.

44. Taylor LO (2003) The hedonic method. In: Champ PA, Boyle KJ, Brown TC, editors. A Primer on Nonmarket Valuation. Dordrecht, Netherlands: Kluwer Academic Publishers. pp. 331-394.

45. Holmes TP (1991) Price and welfare effects of catastrophic forest damage from southern pine beetle epidemics. For Sci 37: 500-516.

46. Prestemon JP, Holmes TP (2000) Timber price dynamics following a natural catastrophe. Am J Agr Econ 82: 145-160.

47. This program is available at http://wwwncrs2fsfedus/4801/fiadb/fim40/wcfim40asp.

48. Minnesota Department of Natural Resources (2009) Minnesota’s ash resource and markets. Minnesota Department of Natural Resources, Forest Products Utilization Program.

49. Wisconsin Department of Natural Resources (2007) Eastern Hemlock. Wisconsin Department of Natural Resources, Forest Products Utilization and Marketing.

50. Wisconsin Department of Natural Resources (2007) Timber harvest in Wisconsin . Wisconsin Department of Natural Resources, Forest Products Utilization and Marketing.

51. These reports can found at http://wwwsrsfsusdagov/econ/data/prices/.

52. Gansner DA, Arner SL, Widmann RH, Alerich. CL (1993) After two decades of gypsy moth, is there any oak left? North J Appl For 10: 184-186.

53. Davidson CB, Gottschalk KW, Johnson JE (1999) Tree mortality following defoliation by the European gypsy moth (*Lymantria dispar* L.) in the United States: A review. For Sci 45: 74-84.

54. Morin RSJ, Liebhold AM, Gottschalk KW (2004) Area-wide analysis of hardwood defoliator effects on tree conditions in the Allegheny Plateau. North J Appl For 21: 31-39.

55. Pimentel D, Lach L, Zuniga R, Morrison D (2000) Environmental and economic costs of nonindigenous species in the United States. Bioscience 50: 53-65.

56. Lovett GM, Canham CD, Arthur MA, Weathers KC, Fitzhugh RD (2006) Forest ecosystem responses to exotic pests and pathogens in eastern North America. Bioscience 56: 395-405.

57. Walsh RG, Bjonback RD, Aiken RA, Rosenthal DH (1990) Estimating the public benefits of protecting forest quality. J Environ Manage 30: 175-189.

58. Kramer RA, Holmes T, Haefele M (2003) Contingent valuation of forest ecosystem protection. In: E. Sills KA, editor. Forests in a Market Economy. Dordrecht, Netherlands: Kluwer Academic Publishers. pp. 303-320.

59. Loomis J, Lockwood M, Delacy T (1993) Some emperical-evidence on embedding effects in contingent valuation of forest protection. J Environ Econ Manage 25: 45-55.

60. Rollins K, Lyke A (1998) The case for diminishing marginal existence values. J Environ Econ Manage 36: 324-344.

61. Aukema JE, McCullough DG, Von Holle B, Liebhold AM, Britton K, et al. (2010) Historical accumulation of nonindigenous forest pests in the continental US BioScience 60: 886-897.

62. Mattson W, Niemelä P, Millers I, Inguanzo Y (1994) Immigrant phytophagous insects on woody plants in the United States and Canada: an annotated list. GTR NC-169. St. Paul, MN: U.S. Department of Agriculture.

63. Mattson W, Vanhanen H, Veteli T, Sivonen S, Niemela P (2007) Few immigrant phytophagous insects on woody plants in Europe: legacy of the European crucible? Biol Invasions 9: 957-974.

64. Williamson M, Fitter A (1996) The varying success of invaders. Ecology 77: 1661-1666.

65. Evans M, Hastings N, Peacock B (1993) Statistical Distributions. New York, NY John Wiley & Sons, Inc.

66. Gilks WR, Richardson S, Spiegelhalter DJ (1996) Markov Chain Monte Carlo In Practice. Boca Raton, Florida: Chapman & Hall/CRC Press.

67. Wintle BA, McCarthy MA, Volinsky CT, Kavanagh RP (2003) The Use of Bayesian Model Averaging to Better Represent Uncertainty in Ecological Models. Conservation Biol 17**:** 1579-1590.

68. Kovacs KF, Haight RF, McCullough DG, Mercader RJ, Siegert NW, Liebhold AM (2011) The influence of satellite populations of emerald ash borer on projected economic costs in U.S. communities, 2010-2020. J Environment Manage 92: 2170-2181.

69. Miles PD, Brand GJ, Alerich CL, Bednar LR, Woudenberg SW, Glover JF, Ezzell EN (2001) The forest inventory and analysis database: database description and users manual version 1.0. General Technical Report NC-218, North Central Research Station, USDA Forest Service, St. Paul, MN.

70. Waters WE, Stark RW (1980) Forest pest management: concept and reality. Annu Rev Entomol 25: 479-509.
