# Supplementary figures and images for: Economic Impacts of Non-Native Forest Insects in the Continental United States
Source: PLoS One. 2011 Sep 9;6(9):e24587. doi: 10.1371/journal.pone.0024587 (PMC3170362; doi:10.1371/journal.pone.0024587)

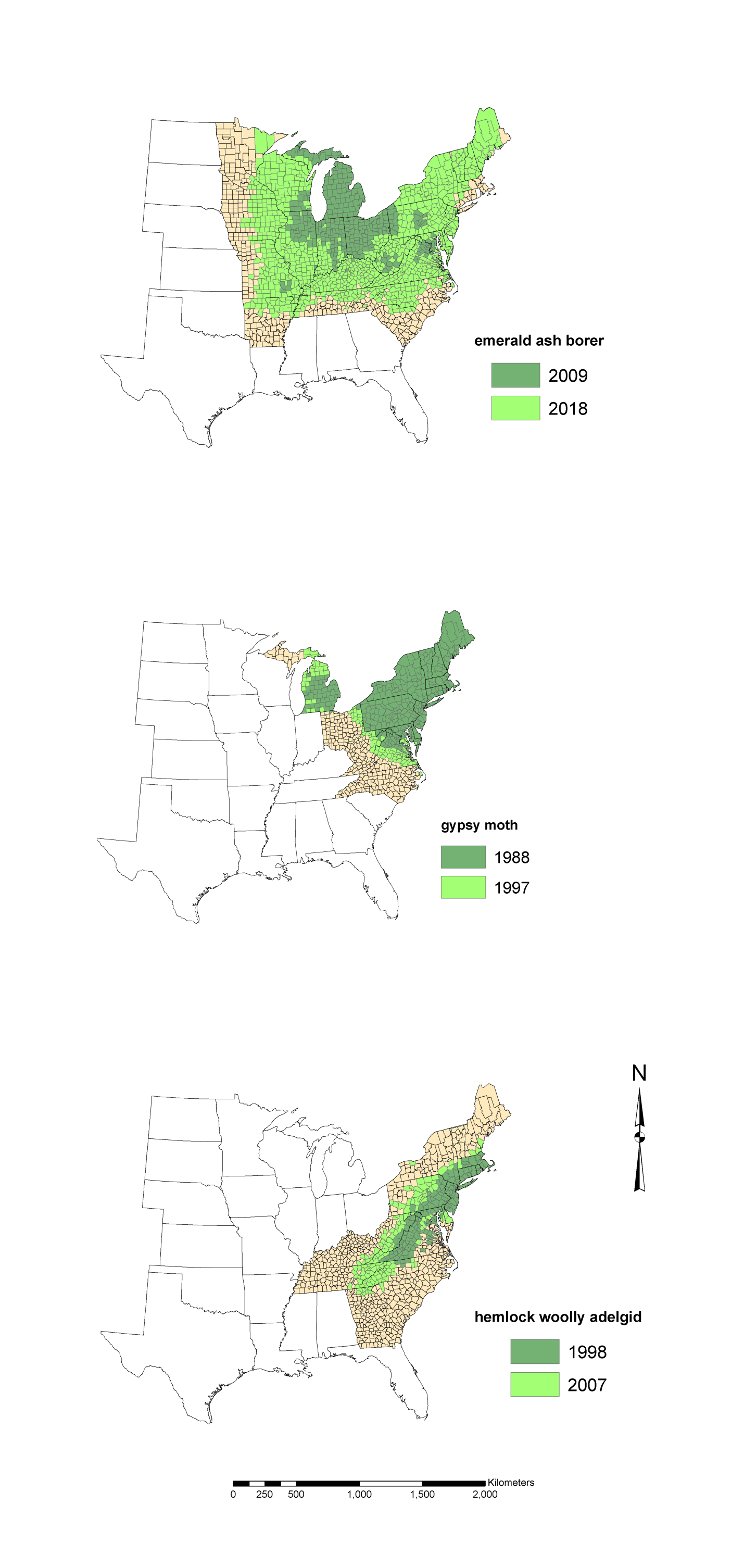

Supplement: Figure S1 — Study area of U.S. counties infested at the beginning of the study period (shaded green) and at the end of the study period (shaded yellow) by the emerald ash borer (top), the gypsy moth (middle), and the hemlock wooly adelgid (bottom). (TIF) [file pone.0024587.s002.tif]

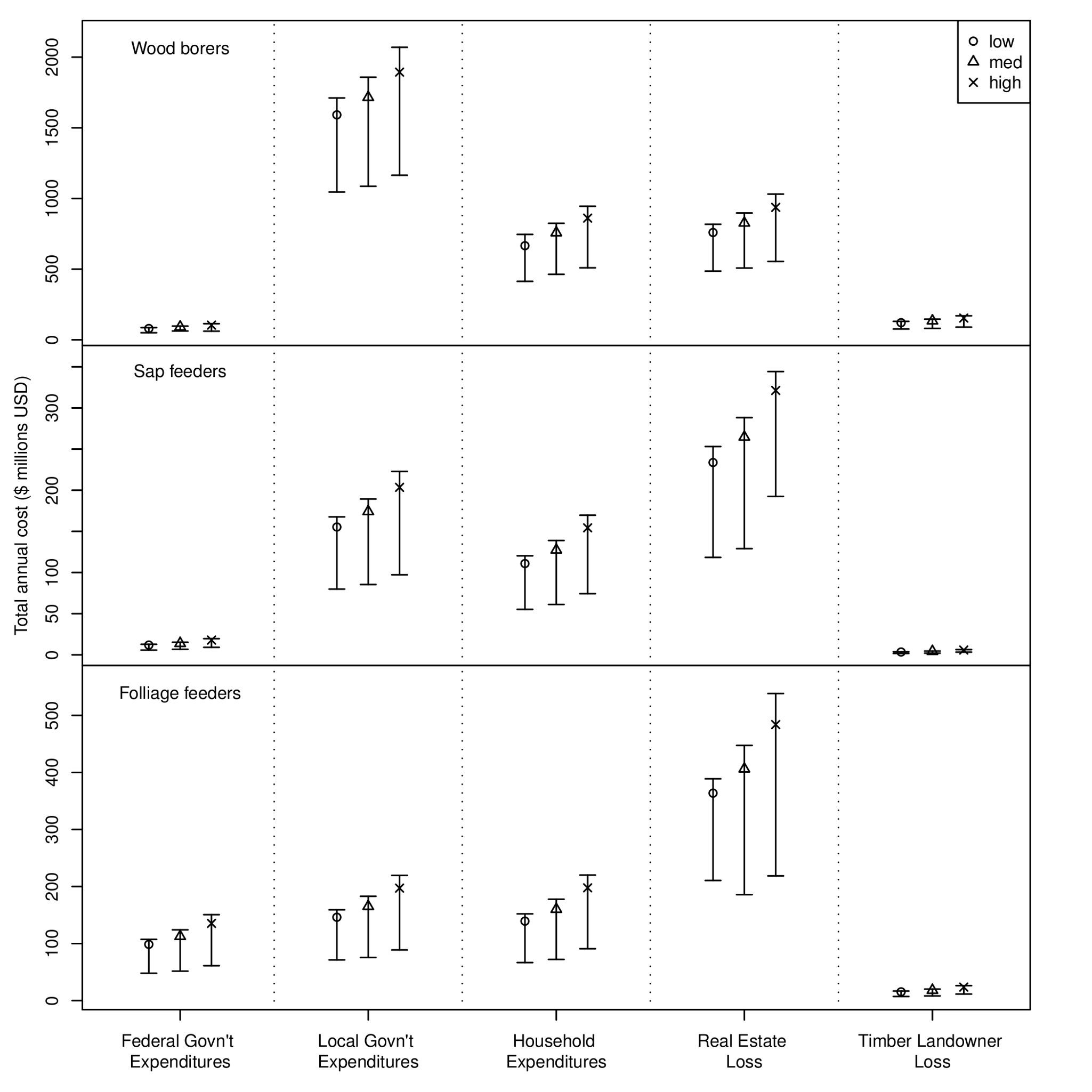

Supplement: Figure S2 — Sensitivity of total cost estimate to the lower threshold for each guild and cost category combination. Low and high represent posterior values obtained using a lower threshold one order of magnitude below and above (respectively) expert opinion (medium). Mean and 90% Bayesian credible intervals illustrated. (TIFF) [file pone.0024587.s003.tiff]

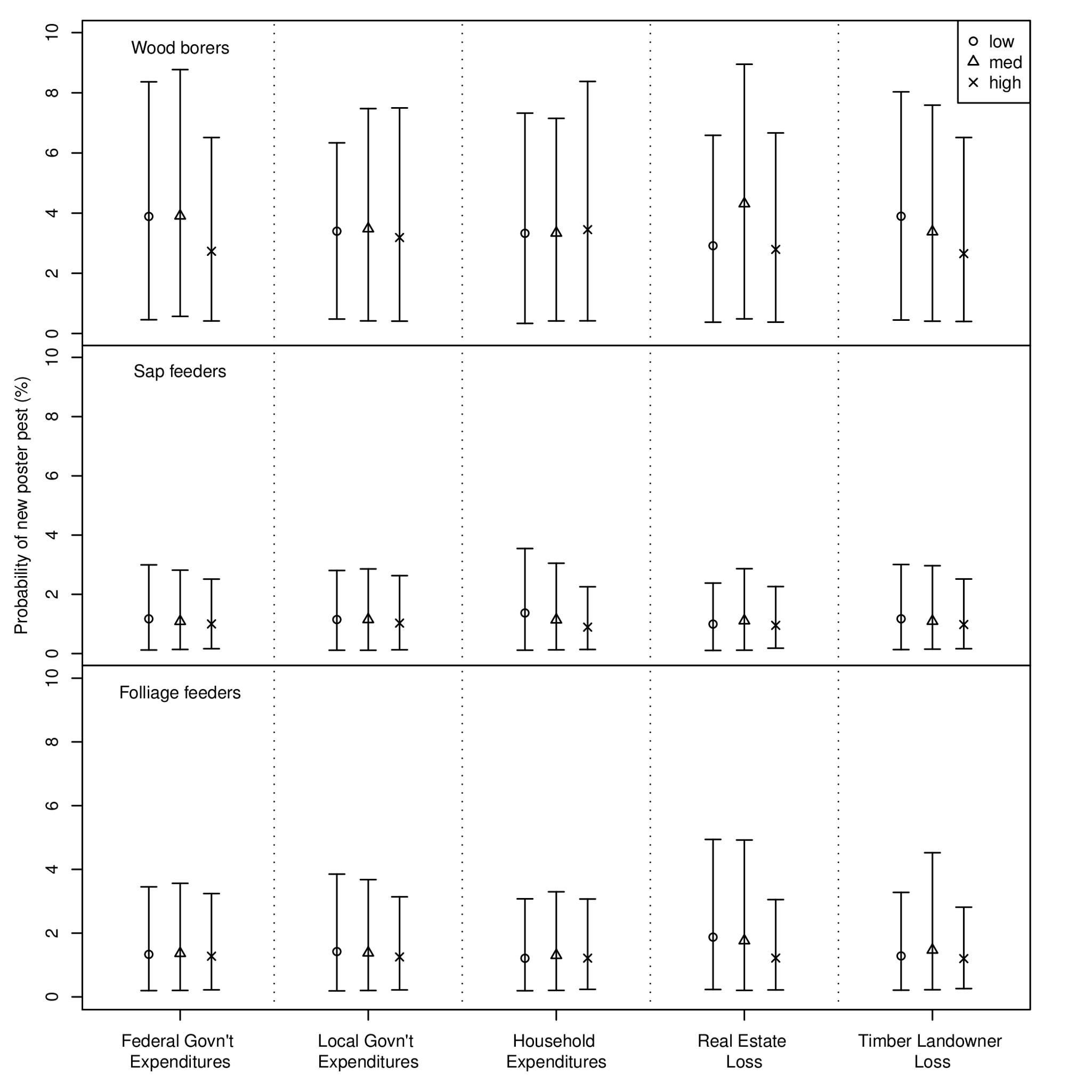

Supplement: Figure S3 — Sensitivity to the lower threshold of the probability of a new pest as damaging or more damaging than the poster pest for each guild and cost category combination. Low and high represent posterior values obtained using a lower threshold one order of magnitude below and above (respectively) expert opinion (medium). Mean and 90% Bayesian credible intervals illustrated for each guild and cost category combination. (TIFF) [file pone.0024587.s004.tiff]
