# Supplementary material for: Economic Impacts of Non-Native Forest Insects in the Continental United States
Source: PLoS One. 2011 Sep 9;6(9):e24587. doi: 10.1371/journal.pone.0024587 (PMC3170362; doi:10.1371/journal.pone.0024587)
Supplement: Table S1 — Ten-year time horizon for calculating poster pest damages. (DOC) [file pone.0024587.s005.doc]

Table S1. Ten-year time horizon for calculating poster pest damages

| Poster Pest | Federal Government Expenditures | Local Government Expenditures | Household Expenditures | Residential Property Value Loss | Forest Landowner Timber Loss |
| --- | --- | --- | --- | --- | --- |
| Emerald ash borer | 2003-2012 | 2009-2018 | 2009-2018 | 2009-2018 | 2009-2018 |
| Hemlock woolly adelgid | 2000-2009 | 1998-2007 | 1998-2007 | 1998-2007 | 1998-2007 |
| Gypsy moth | 1998-2007 | 1988-1997 | 1988-1997 | 1988-1997 | 1988-1997 |
