# Supplementary material for: Economic Impacts of Non-Native Forest Insects in the Continental United States
Source: PLoS One. 2011 Sep 9;6(9):e24587. doi: 10.1371/journal.pone.0024587 (PMC3170362; doi:10.1371/journal.pone.0024587)
Supplement: Table S2 — Host tree density on developed land for the study areas corresponding to the emerald ash borer and hemlock woolly adelgid. (DOC) [file pone.0024587.s006.doc]

Table S2. Host tree density on developed land for the study areas corresponding to the emerald ash borer and hemlock woolly adelgid.

| Average city inventory | Emerald ash borer^1^ | Hemlock woolly adelgid^2^ |
| --- | --- | --- |
| Developed land (ha) | 18,594 | 12,092 |
| Canopy cover (ha) | 2,279 | 2,321 |
| Host trees per ha developed land | 6.25 | 7.82 |
| Host trees per ha canopy cover | 86.53 | 69.30 |

^1^ Cities of Atlanta, GA; Baltimore, MD; Boston, MA; Chicago, IL; Indianapolis, IN; Livonia, MI; Milwaukee, WI; Minneapolis, MN; Morgantown, WV; Palatine, IL; Philadelphia, PA; Syracuse, NY; Troy, MI; Urbana, IL; Washington, DC; Wilmington, DE.

^2^ Cities of Boston, MA; Jersey City, NJ; Philadelphia, PA; Baltimore, MD; Atlanta, GA; Syracuse, NY
