# Supplementary material for: Economic Impacts of Non-Native Forest Insects in the Continental United States
Source: PLoS One. 2011 Sep 9;6(9):e24587. doi: 10.1371/journal.pone.0024587 (PMC3170362; doi:10.1371/journal.pone.0024587)
Supplement: Table S3 — Ash density by land use and diameter class for the city of Chicago. (DOC) [file pone.0024587.s007.doc]

Table S3. Ash density by land use and diameter class for the city of Chicago.

| Land use | Percent of | Ash trees | Ash trees per ha cover by diameter class | | |
| --- | --- | --- | --- | --- | --- |
|  | urban land | per ha cover | 0-30 cm | 30-61 cm | > 61 cm |
| Residential | 0.64 | 141.7 | 47.5 | 34.6 | 8.6 |
| Non-residential | 0.36 | 551.9 | 177.1 | 17.3 | 4.3 |
