# Supplementary material for: Economic Impacts of Non-Native Forest Insects in the Continental United States
Source: PLoS One. 2011 Sep 9;6(9):e24587. doi: 10.1371/journal.pone.0024587 (PMC3170362; doi:10.1371/journal.pone.0024587)
Supplement: Table S4 — Management costs for homeowners and community managers. (DOC) [file pone.0024587.s008.doc]

Table S4. Management costs for homeowners and community managers.

| Landowner | Costs ($/tree) | | | |
| --- | --- | --- | --- | --- |
|  | Remove | Remove and replace | Ash  Treatment | Hemlock  Treatment |
|  | --------------- Tree size = 0-30 cm in DBH ------------- | | | |
| Homeowner | 200 | 600 | 54 | 40 |
| Community | 150 | 450 | 50 | 16 |
|  | --------------- Tree size = 30-61 cm in DBH ------------ | | | |
| Homeowner | 400 | 800 | 120 | 89 |
| Community | 300 | 600 | 100 | 32 |
|  | --------------- Tree size = >61 cm in DBH -------------- | | | |
| Homeowner | 1100 | 1500 | 200 | 148 |
| Community | 900 | 1200 | 150 | 48 |

Note: Cost estimates for the remove and replacement of ash and hemlock trees, and the ash treatment come from the EAB cost calculator (http://www.entm.purdue.edu/EAB/). Cost estimates for the hemlock treatment are from Cowles et al. [36].
