# Supplementary material for: Economic Impacts of Non-Native Forest Insects in the Continental United States
Source: PLoS One. 2011 Sep 9;6(9):e24587. doi: 10.1371/journal.pone.0024587 (PMC3170362; doi:10.1371/journal.pone.0024587)
Supplement: Table S5 — Parameters for the model of timber losses to forest landowners. (DOC) [file pone.0024587.s009.doc]

Table S5. Parameters for the model of timber losses to forest landowners.

|  | Emerald ash borer | Hemlock woolly adelgid | Gypsy moth |
| --- | --- | --- | --- |
| Timber Species | Ash | Eastern Hemlock | Oak |
| Proportion of harvest  Sawtimber  Pulpwood  Fuelwood | 0.5  0.25  0.25 | 0.79  0.21  0 | 0.45  0.25  0.30 |
| Stumpage Price ($/cubic foot)  Sawtimber  Pulpwood  Fuelwood | 0.77  1.46  0.08  0.09 | 0.28  0.53  0.07  0 | 1.18  2.24  0.09  0.14 |
| Years of harvest loss | 10 10 10 | | |
