# Supplementary material for: Economic Impacts of Non-Native Forest Insects in the Continental United States
Source: PLoS One. 2011 Sep 9;6(9):e24587. doi: 10.1371/journal.pone.0024587 (PMC3170362; doi:10.1371/journal.pone.0024587)
Supplement: Table S6 — Lower economic threshold of damages by damage category. (DOC) [file pone.0024587.s010.doc]

Table S6. Lower economic threshold of damages by damage category

| Damage Category | Lower economic threshold  (annual damages) | Justification |
| --- | --- | --- |
| Federal and Local Government Expenditures | $1,500 | One FHP person  3 days/year |
| Household Expenditures | $4,000 | 200 households spend $20/year |
| Residential Property Value Loss | $4,000 | 200 households spend $20/year |
| Forest Landowner Timber Loss | $1,500 | One FHP person  3 days/year |
